# Intravital imaging of Wnt/β-catenin and ATF2-dependent signalling pathways during tumour cell invasion and metastasis

**DOI:** 10.1242/jcs.260285

**Published:** 2023-02-10

**Authors:** Konstantin Stoletov, Saray Sanchez, Irantzu Gorroño, Miriam Rabano, Maria d. M. Vivanco, Robert Kypta, John D. Lewis

**Affiliations:** ^1^ Department of Oncology, University of Alberta, Edmonton, Alberta T6G 2E1, Canada; ^2^ Centre for Cooperative Research in Biosciences (CIC bioGUNE), Basque Research and Technology Alliance (BRTA), Bizkaia Technology Park, Building 801A, 48160 Derio, Spain; ^3^ Department of Surgery and Cancer, Imperial College London, London W12 0NN, UK

**Keywords:** Wnt, *In vivo* imaging, Cancer metastasis

## Abstract

Wnt signalling has been implicated as a driver of tumour cell metastasis, but less is known about which branches of Wnt signalling are involved and when they act in the metastatic cascade. Here, using a unique intravital imaging platform and fluorescent reporters, we visualised β-catenin/TCF-dependent and ATF2-dependent signalling activities during human cancer cell invasion, intravasation and metastatic lesion formation in the chick embryo host. We found that cancer cells readily shifted between states of low and high canonical Wnt activity. Cancer cells that displayed low Wnt canonical activity showed higher invasion and intravasation potential in primary tumours and in metastatic lesions. In contrast, cancer cells showing low ATF2-dependent activity were significantly less invasive both at the front of primary tumours and in metastatic lesions. Simultaneous visualisation of both these reporters using a double-reporter cell line confirmed their complementary activities in primary tumours and metastatic lesions. These findings might inform the development of therapies that target different branches of Wnt signalling at specific stages of metastasis.

## Teaser

**Summary:** Using intravital imaging and an avian embryo xenograft model, we show that canonical Wnt and ATF2 signalling pathways have complementary activity patterns during human cancer cell invasion and metastasis.

## INTRODUCTION

Human cancers often exploit signalling pathways that are involved in normal tissue growth and organ development. Wnt signalling is one of the best-known examples of a developmental pathway that is commonly hijacked by cancer cells. During normal development, Wnt signals control multiple processes, including stem cell maintenance, cell polarity and individual and collective cell migration (Steinhart and Angers, 2018). Similarly, when utilised by cancer cells, Wnt signals participate in a variety of processes that drive cancer progression (Zhan et al., 2017; Nusse and Clevers, 2017).

Canonical Wnt signalling operates via activation of β-catenin-TCF/LEF (T cell factor/lymphoid enhancer factor family)-dependent gene transcription and is mainly implicated in stem cell maintenance and cell proliferation (Nusse and Clevers, 2017). The cancer stem cell (CSC) model proposes that only a small percentage of cancer cells can establish secondary lesions, and this has increased interest in the role of this branch of Wnt signalling in tumour metastasis. Indeed, cancer cells with augmented canonical Wnt activity (cWnt^high^) *in vitro* show increased capacity for tumour initiation *in vivo* (Vermeulen et al., 2010). Throughout the metastatic cascade, cWnt^high^ cells are often found in locations associated with CSC function (Vermeulen et al., 2010; Malanchi et al., 2011). In early stages of colon cancer, for example, cWnt^high^ cells reside at the base of crypts in close association with myofibroblasts, which secrete factors capable of further activating Wnt signalling (Aizawa et al., 2019). In breast cancer, cWnt^high^ cells can be preferentially found away from hypoxic areas and in the vicinity of the vasculature, suggesting that these cells can intravasate and metastasise more readily (Vadakkan et al., 2014). Metastatic lung adenocarcinoma cells isolated from lymph nodes have increased TCF/LEF activity, suggesting again that cWnt^high^ cells are responsible for the initial steps of metastasis, such as invasion (Nguyen et al., 2009). Upon arrival at the metastatic site, CSCs continue to interact with the vasculature, where they secrete stromal factors that maintain active Wnt/TCF signalling and lead to further expansion of metastatic lesions (Malanchi et al., 2011; Esposito et al., 2019). Moreover, at later stages of metastasis, TCF-driven gene transcription is necessary for organ (brain and bone)-specific colonisation (Vadakkan et al., 2014). Finally, multiple reports directly implicate the Wnt/β-catenin signalling arm in driving cancer cell invasion. Indeed, in colon cancer, SNAIL1 (also known as SNAI1) is capable of fine-tuning Wnt/β-catenin signalling to promote invasion and proliferation (Freihen et al., 2020). In glioblastoma, cWnt^high^ cells can attach and invade more efficiently along the vasculature, which is the main mechanism of glioblastoma expansion (Griveau et al., 2018). Moreover, suppression of β-catenin expression has recently been reported to reduce *in vitro* migration and invasion in head and neck squamous cell carcinoma (HNSCC) model (Moon et al., 2021).

However, other recent findings cast doubt on the straightforward role of Wnt/β-catenin signalling as a driver of cancer cell tumour initiating and invasive properties. In a mouse xenograft model of colon cancer, cWnt^high^ cells were shown to have only a marginal increase in their tumour-initiating properties. Genetic interference with Wnt/β-catenin signalling was found to increase colon cancer cell invasive potential, contrary to what was expected. In a patient setting, the activation of the Wnt/β-catenin–TCF axis was found to be significantly lower in the CD133^+^ colon CSC-like population and its inhibition led to a boost in tumour growth in mouse xenografts (Horst et al., 2012; Chen et al., 2021b; Varnat et al., 2010; Kramer et al., 2017).

Less is known about the role of noncanonical (β-catenin-independent) Wnt signalling during cancer cell metastasis, yet the consensus is that it promotes cancer cell invasion in a broad range of human cancers when activated (VanderVorst et al., 2019; Chen et al., 2021a,b), including melanoma (Weeraratna et al., 2002), ovarian (Asad et al., 2014), gastric (Kurayoshi et al., 2006) and pancreatic (Yu et al., 2012) cancers. During development, certain Wnts activate planar cell polarity (PCP) signalling, which involves activation of Rho, Rac and JNK proteins, and leads to activation of the transcription factor ATF2, among others (Schambony and Wedlich, 2007; Zhou et al., 2007). This branch of Wnt signalling can be measured in *Xenopus* embryos using an ATF2-dependent gene reporter (Ohkawara and Niehrs, 2011). The use of ATF2-dependent reporters in mammalian cells is controversial, not least because ATF2-dependent transcription can also be activated during the cell stress response (Yamamoto et al., 2008). Nevertheless, they have been used to measure responses to Wnt-11 in prostate and colorectal cancer cells (Murillo-Garzón et al., 2018; Gorroño-Etxebarria et al., 2019) and in chick embryo mesenchymal cells (Boitard et al., 2015), to Wnt-5a in osteosarcoma cells (Yamagata et al., 2012) and to collagen triple helix repeat containing-1, a protein that enhances Wnt interaction with FZD proteins and/or ROR2 (Zhang et al., 2017) in primary gastrointestinal stromal tumour (GIST) cells (Gorroño-Etxebarria et al., 2019). Adding to the complexity, there is signalling crosstalk between noncanonical and canonical Wnt signalling, with priming of noncanonical signalling by canonical signalling and inhibition of canonical signalling by noncanonical signalling (Watson et al., 2017; Ma et al., 2022; Maye et al., 2004; Park et al., 2015).

Together, these studies suggest that the spatio-temporal map of β-catenin/TCF-dependent and ATF2-dependent signalling activation during cancer progression is likely to be complex. During the metastatic cascade, Wnt signals might be activated at distinct locations and at different times within the same tumour, metastatic lesion, and even in individual cancer cells. Until recently, continuous intravital visualisation of Wnt pathway activation during cancer progression has not been possible. Here, we use fluorescent β-catenin/TCF-dependent and ATF2-dependent reporters as tools to visualise their activities during cancer cell invasion, intravasation and metastatic lesion formation. We show that canonical Wnt signalling is mostly downregulated during all these steps and is restricted to a limited population of cells with low motility. In contrast ATF2-dependent signalling is active in most invasive cells, both in primary tumours and in metastatic lesions. Throughout the metastatic cascade β-catenin/TCF-dependent and ATF2-dependent signalling pathways appear to complement one another, being active in distinct zones within the primary tumour and in metastatic lesions.

## RESULTS

### Canonical Wnt pathway reporter activity is downregulated during primary tumour cell invasion

Although fluorescent reporters have been used in a variety of *in vivo* systems to study Wnt signalling activity during development (Rios et al., 2010; Ferrer-Vaquer et al., 2010) and in tumour xenografts (Vadakkan et al., 2014; Kim et al., 2019), visualisation of Wnt pathway activity at the cellular level during metastasis has not yet been described. Recently, we developed a unique avian embryo model of human cancer metastasis that allows for high-resolution intravital imaging of cancer cell invasion, intravasation, extravasation and metastatic lesion formation (Stoletov et al., 2018; Leong et al., 2014; Ngan et al., 2017; Zijlstra et al., 2008). Importantly, our model permits continuous visualisation of invasive cancer cell behaviour and cancer cell interaction with the vasculature and extracellular matrix both in the primary tumour and metastatic lesions (Stoletov et al., 2018; Leong et al., 2014; Ngan et al., 2017).

**Fig. 1.**
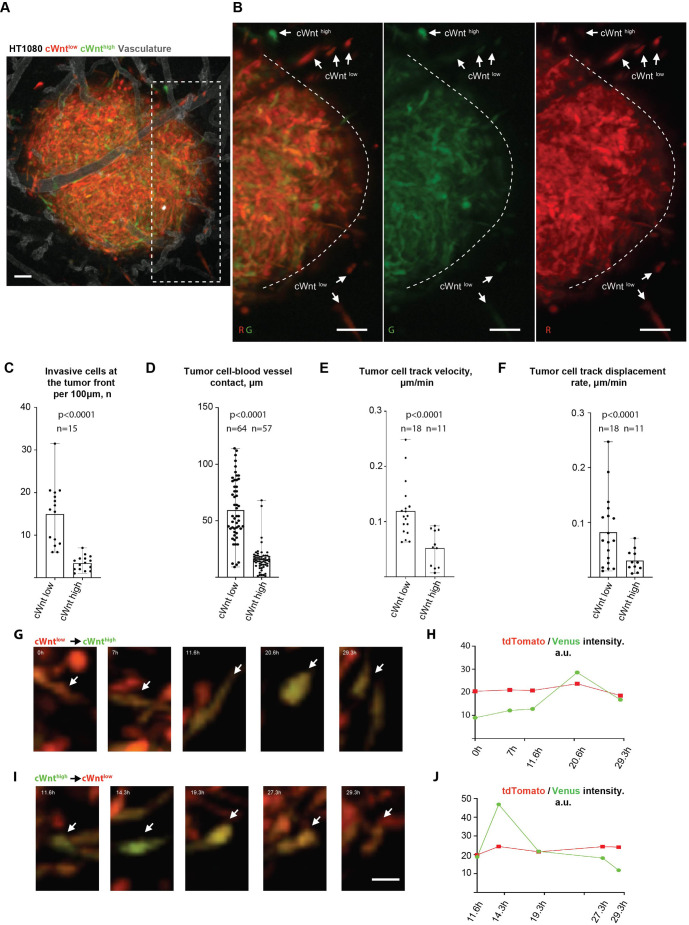
**Canonical Wnt pathway activity is downregulated during primary tumour invasion.** (A) Representative image showing overall canonical pathway Wnt reporter activity pattern during HT1080 primary tumour invasion. (B) Images showing red/green (tdTomato and Venus), green only and red only channels for the invasive front area from within the dashed square in A. Examples of cancer cells that display high (cWnt^high^) or low (cWnt^low^) canonical Wnt pathway activity are highlighted by white arrowheads. Dashed line highlights the tumour front. (C) Quantification of cWnt^low^ and cWnt^high^ cancer cell number at the primary tumour invasive front. (D) Quantification of the mean tumour cell–blood vessel contact length formed by cWnt^low^ or cWnt^high^ HT1080 cancer cells at the tumour invasive front. (E) Quantification of mean cWnt^low^ and cWnt^high^ cancer cell track velocities. (F) Quantification of the mean cWnt^low^ and cWnt^high^ cancer cell track displacement rates. Results are mean with error bars showing the complete range of results. (G–J) Time-series images showing examples of cWnt^low^ to cWnt^high^ and cWnt^high^ to cWnt^low^ transitions (G,I) and individual (average per cell area) channel intensity measurements (H,J) at the invasive tumour front (arrows; also see Movie 1). au, arbitrary units. Statistical comparison [unpaired two-tailed *t*-test (E) or Mann–Whitney test (C,D,F)]. *n* animals=15 (C,D) and 5 (E,F). Scale bars: 50 µm (A,B); 20 µm (G,I).

To visualise canonical Wnt pathway activity during cancer progression we generated HT1080 human fibrosarcoma cells genetically engineered to express β-catenin/TCF-activated Venus fluorescent protein (pBARV, Biechele and Moon, 2008; here, called TOP-Venus) in a background of constitutively expressed tdTomato (Fig. 1A; Fig. S1A,B). Control cells expressed fuBARV (Biechele and Moon, 2008; here, called FOP-Venus), which is pBARV with mutations in the TCF/LEF-binding sites. We chose HT1080 cells because they robustly form primary tumours and metastasise in the avian embryo model, allowing for monitoring of all the steps of metastatic cascade. HT1080 TOP-Venus cells were sorted by FACS to select cells with a robust response to activators of Wnt signalling. Analysis of the *in vitro* responses to treatment with recombinant Wnt-3a protein, Wnt-3a conditioned medium and the GSK-3 inhibitor CHIR99021 found that the vast majority of HT1080 TOP-Venus cells showed strong Venus fluorescence within 12 h of stimulation, and the activation was strong for 24–48 h, with unaltered expression of TdTomato and; in contrast, HT1080 FOP-Venus cells showed no detectable responses to Wnt-3a or CHIR99021 *in vitro* (Fig. S1A–D).

To model primary tumour invasion, HT1080 TOP-Venus cells were inoculated onto the chorioallantoic membrane (CAM) as previously described (Stoletov et al., 2018). In this setting, HT1080 cells form rapidly growing tumours that are highly invasive and angiogenic (Stoletov et al., 2018; Leong et al., 2014; Ngan et al., 2017; Deryugina and Kiosses, 2017). Interestingly, most of the cells at the invasive tumour front displayed low canonical Wnt pathway reporter activity (cWnt^low^; green to red signal average ratio <1.5) with cWnt^low^ cells constituting the majority (>80%) of the cells at the invasive tumour zone (Fig. 1A–C). In contrast, the majority of cells with high canonical pathway reporter activity (cWnt^high^; green to red signal average ratio >1.5) was located within the main tumour mass (Fig. 1A–C). HT1080 FOP-Venus control cells did not show any significant Venus fluorescence *in vivo* (Fig. S1E). At the invasive tumour front, cWnt^high^ cells were more rounded (Fig. S2A) and formed shorter contacts with the vasculature when compared to what was seen for cWnt^low^ cells (Fig. 1D), suggesting that cWnt^high^ cells have a decreased potential for perivascular invasion. Tracking analysis showed that cWnt^high^ cells invaded significantly more slowly and in a less directional manner than cWnt^low^ cells (Fig. 1E,F; Movie 1). In accordance with these results, exogenous activation of Wnt/β-catenin with recombinant Wnt-3a protein and CHIR99021 reduced migration of HT1080 cells in *in vitro* t-scratch assays (Fig. S2B–E). The finding that cWnt^high^ HT1080 cells were less invasive was surprising, as we were expecting that even if cWnt^high^ cells constituted a minority (5.6±3.6%; mean±s.d.) population in the primary tumour, they would still be responsible for most of the invasion and metastatic dissemination, as suggested previously (Vadakkan et al., 2014).

Surprisingly, during long term time-lapse analysis of cancer cell invasion at the primary tumour front, we found that invasive HT1080 cells readily switched between cWnt^low^ and cWnt^high^ states (Fig. 1G,I; Movie 1). These switches were not due to changes in tdTomato fluorescence intensity, but to the changes in Venus fluorescence (driven by the TCF/LEF-binding sites in pBARV) and could occur within a relatively short (∼12 h) period of observation (Fig. 1H,J; Movie 1).

**Fig. 2.**
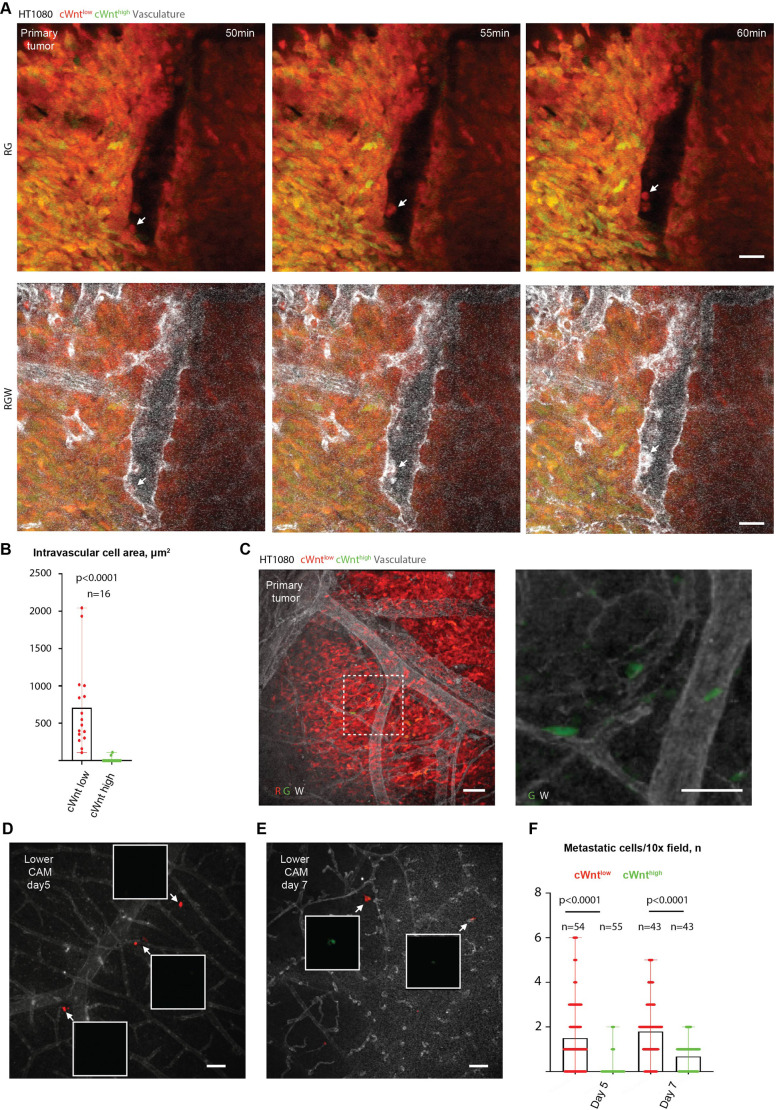
**Canonical Wnt pathway reporter activity decreases during the initial stages of spontaneous HT1080 cancer cell metastasis.** (A) Time-lapse images (maximum intensity projection) showing cWnt^low^ cell intravasating and rolling along the vascular wall within the primary tumour mass (white arrow). Also see the Movie 2 for the complete time-lapse video of the same field. (B) Quantification of cWnt^high^ and cWnt^low^ cells protrusion into the vascular lumen. (C) Representative image showing canonical Wnt pathway reporter activity pattern in a HT1080 tdTomato (R, red) and TOP Venus (G, green) primary tumour (*in ovo* spontaneous metastasis assay, maximum intensity projections). Vasculature is stained with Lectin 647 (W, white). Right panel shows Venus (G) and Lectin 647 channels (W) only from within the dashed square. (D,E) Representative images of random lower CAM fields 5 (b) and 7 (c) days post tumour inoculation (maximum intensity projections). Insets show Venus channel only in the cells highlighted by white arrows. (F) Quantification of cWnt positive (Venus^+^) HT1080 metastatic colonies 5 and 7 days post-tumour inoculation. Statistical comparison was performed using Mann–Whitney test. *n* animals=16 (B); 13 (day 5) and 16 (day 7, F). Results in B and F are mean with error bars showing the complete range of results. Scale bars: 50 µm.

Recent evidence suggests that the majority of cancer cell intravasation occurs within the tumour mass and that cancer cells that invade out of the primary tumour are not responsible for the bulk of metastatic dissemination (Deryugina and Kiosses, 2017). Given that our model permits live imaging of cancer cell interactions with the vasculature, we assessed whether cWnt^high^ cells were responsible for the majority of the intravasation and early stages of metastatic lesion dissemination of tumour cells. Although relatively rare, intravasation events can be routinely observed in this model when cancer cells protrude into the vessel lumen and are carried away by the flow of the blood. First, detailed visualisation of the vasculature at the primary tumour front was surveyed in order to detect intravasation events. This found that in all the cases observed (>30), all the cells that were completely intravasated or rolling along the vascular endothelium or carried by the blood flow were cWnt^low^ (Movie 1, inset, and Movie 2). High-resolution visualisation of cancer cell interaction with the tumour vasculature showed that, even though in some cases cWnt^high^ cells were observed to the contact vascular wall, their ability to protrude into the vascular lumen was significantly reduced when compared to that of cWnt^low^ cells (Fig. 2A,B; Movie 2). To further study this behaviour, we employed an *in ovo* spontaneous metastasis assay, where primary tumours are grown on the upper CAM within the uncracked egg and cancer cells are allowed to metastasise spontaneously to the lower CAM and to other organs via the vasculature (Palmer et al., 2011). In this scenario, metastatic cancer cells can be visually detected in the lower CAM as early as 5 days after tumour cell inoculation. Indeed, even if cWnt^high^ cells formed shorter contacts with the vasculature, the majority (>95%) of cWnt^high^ cells were located in the contact with the vasculature within the primary tumours grown in *in ovo* or *ex ovo* (Figs 1A,D and 2C). However, the vast majority of cells detected in the lower CAM at days 5 and 7 after tumour cell inoculation were cWnt^low^ (Fig. 2D–F).

**Fig. 3.**
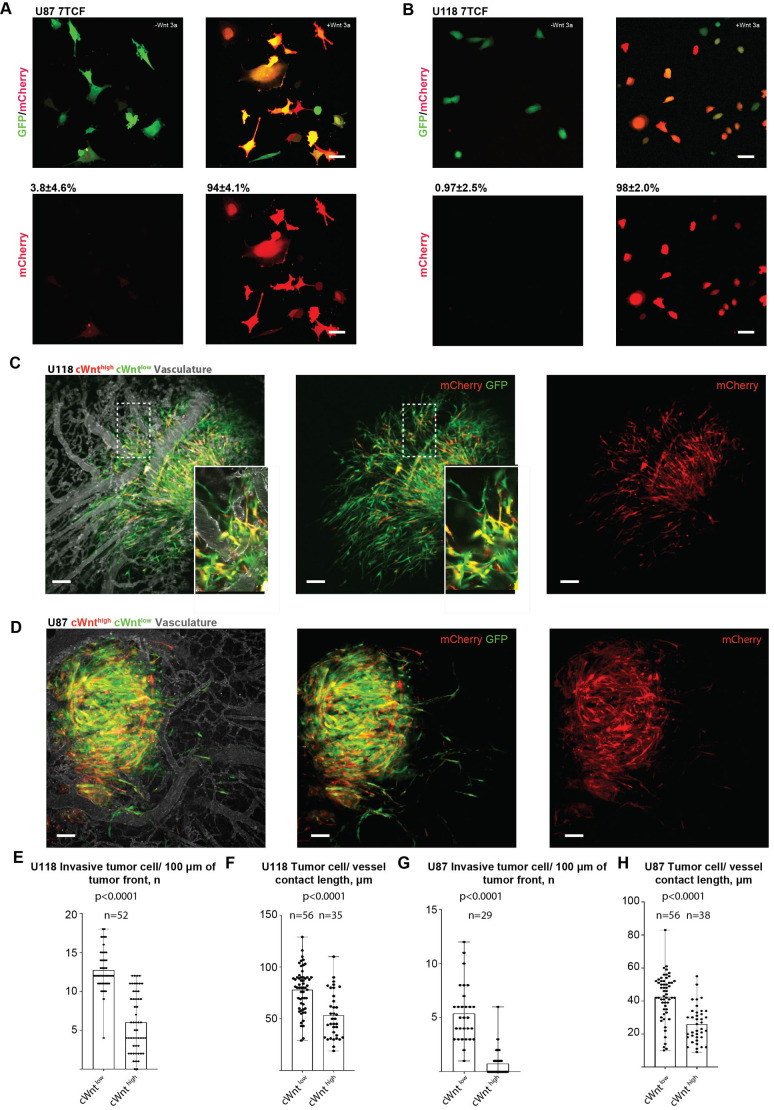
**Canonical Wnt pathway activity is downregulated during glioblastoma cell invasion.** (A,B) Wnt-3a ligand stimulation (100 ng/ml) of U87 7TCfmCherry/GFP (A) and U118 7TCFmCherry/GFP (B) cancer cells. Numbers above the panels show the mean±s.d. (*n*=10) percentage of Wnt-3a reporter-positive cancer cells. (C) Representative images showing the pattern of canonical Wnt reporter activity within primary tumours formed by U118 glioblastoma cells (5 days post-injection, 7TCF-mCherry reporter). (D) Representative images showing the pattern of canonical Wnt reporter activity within primary tumours formed by U87 glioblastoma cells (5 days post-injection, 7TCF-mCherry reporter). (E) Quantification of cWnt^low^ and cWnt^high^ U118 cells at the primary tumour invasive front at 5 days post-tumour cell injection (F) Quantification of mean tumour cell blood vessel contacts that are established by U118 cWnt^low^ or cWnt^high^ cells. (G) Quantification of cWnt^low^ and cWnt^high^ U87 cells at the primary tumour invasive front 5 days post-tumour cell injection. (H) Quantification of mean tumour cell blood vessel contacts established by U87 cWnt^low^ or cWnt^high^ cells. Statistical comparison [unpaired two-tailed *t*-test (F,H) or Mann–Whitney test (E,G)]. *n* animals=5 (E,F) and 6 (G,H). Results in E–H are mean with error bars showing the complete range of results. Scale bars: 50 µm.

Finally, to confirm that the results are not restricted to the HT1080 cell line or one type of cancer, we visualised canonical Wnt pathway activity in invading human glioblastoma cells, chosen because canonical Wnt signalling has been strongly implicated in glioblastoma progression and several therapeutic approaches that target Wnt signalling are currently being explored in this context (Latour et al., 2021; Shahcheraghi et al., 2020). To this purpose, U118 and U87 human glioblastoma cell lines that express the Wnt/β-catenin-responsive reporter 7TCF-mCherry (Fuerer and Nusse, 2010) in a stable GFP expression background were generated. This fluorophore pair was found to produce the brightest fluorescence, allowing for more efficient glioblastoma cell inoculation into the CAM. *In vitro* analysis showed that 7TCF-mCherry U118 and U87 cells uniformly responded to Wnt stimulation (Fig. 3A,B). U118 and U87 7TCF-mCherry reporter cells were inoculated into the chicken CAM and invasive fronts of primary tumours visualised. cWnt^low^ cells dominated the invasive fronts of tumours by both cell lines (Fig. 3C,D), with only ∼30% and ∼15% of U118 and U87 cells at the invasive front, respectively, being cWnt^high^ (Fig. 3E,G). Like HT1080 cells, cWnt^low^ cells of both cell lines displayed more extended contacts with the vasculature (Fig. 3F,H). In contrast, in both glioblastoma lines, cWnt^high^ cells were mostly concentrated in the main tumour mass and displayed reduced vasculotropism (Fig. 3C–H).

### Canonical Wnt pathway reporter activity is downregulated during cancer cell invasion in metastatic lesions

**Fig. 4.**
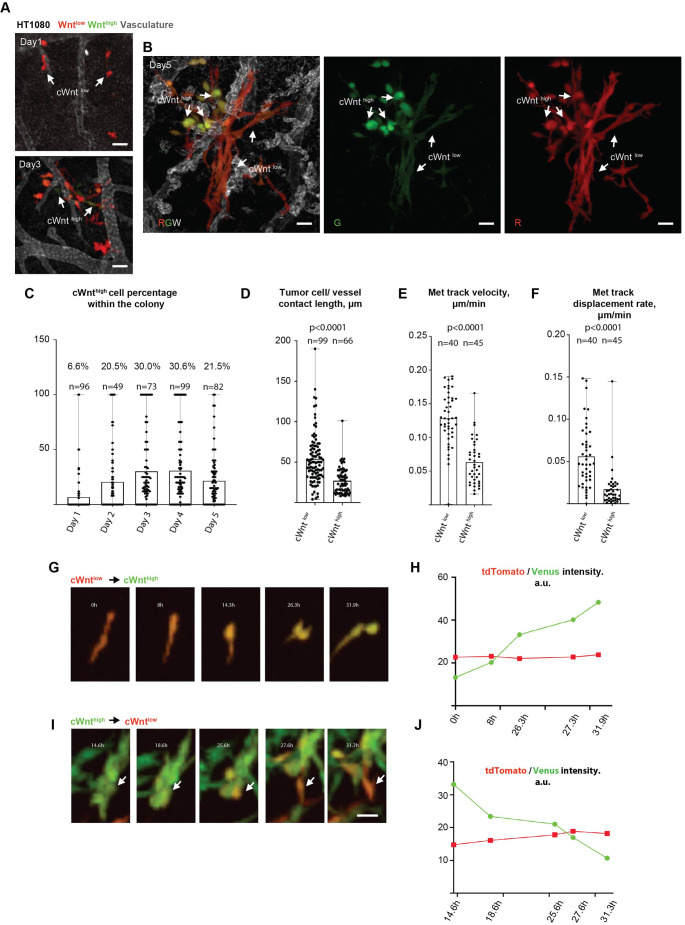
**Canonical Wnt pathway activity is downregulated during invasion of metastatic lesions.** (A) Representative images showing canonical Wnt activity patterns in metastatic lesions formed by HT1080 cells at 1- and 3 days post-injection. (B) Representative images showing canonical Wnt activity patterns within metastatic lesions formed by HT1080 cells at 5 days post-injection (5 dpi). Middle and right-most panels show red/green/white (tdTomato/Venus/Lectin-647); green only and red only channels in A. Examples of cancer cells that display high (cWnt^high^) or low (cWnt^low^) canonical Wnt pathway activity are highlighted by white arrows. (C) Quantification of the percentage of HT1080 cells with increased canonical Wnt pathway activity (cWnt^high^) within the metastatic cancer cell lesions at 1−5 dpi. (D) Quantification of the mean length of tumour cell–blood vessel contacts formed by cWnt^low^ or cWnt^high^ HT1080 cells in metastatic lesions at 5 dpi. (E) Quantification of mean cWnt^low^ and cWnt^high^ cancer cell track velocity during invasion of metastatic cancer cell lesions. (F) Quantification of the average cWnt^low^ and cWnt^high^ cancer cell track displacement rate during the invasion of metastatic cancer cell lesions. Results in C–F are mean with error bars showing the complete range of results. (G–J) Time-series images showing cWnt^low^ to cWnt^high^ and cWnt^high^ to cWnt^low^ (G,I) transitions and individual (average per cell area) channel intensity measurements (H,J) during the invasion of metastatic cancer cell lesions (also see Movie 3). a.u., arbitrary units. Statistical comparison [unpaired two-tailed *t*-test (D) or Mann–Whitney test (E,F)]. *n* animals=at least 12 for every time point in (C); 14 (D) and 6 (E,F). Results in G–J representative of three repeats. Scale bars: 50 µm (A,B) and 20 µm (I, images in G are shown at the same scale).

We next visualised localisation and timing of canonical Wnt pathway reporter activation during metastatic lesion formation. To this purpose, we injected HT1080 TOP-Venus cells intravenously (i.v.; experimental metastasis) and imaged metastatic colony formation within the CAM tissue daily for 5 consecutive days after tumour cell injection (time series analysis; Fig. 4A,B). Interestingly, we found that only a small percentage (6.6%) of HT1080 cells that underwent extravasation and survived 12 h after injection were cWnt^high^ (Fig. 4A,C). During the next 3 days, the proportion of cWnt^high^ cells increased, yet the majority of cells within the metastatic colonies were still cWnt^low^ (Fig. 4C). On the last day of observation (day 5), the proportion of cWnt^high^ cells decreased (from 30.6% to 21.5%) with the majority of the colonies comprising a mixed population (cWnt^high^ and cWnt^low^), although there were also colonies consisting entirely of cWnt^high^ and cWnt^low^ cells. Interestingly, similar to what was observed in primary tumours, we noticed a striking difference in the behaviour of cWnt^high^ and cWnt^low^ HT1080 cells within metastatic lesions (Fig. 4A–F; Movie 3). HT1080 cWnt^low^ cells were significantly more invasive, displaying increased vasculotropism, cell track velocity and displacement rates (Fig. 4B,D–F; Movie 3). In contrast, cWnt^high^ cells were less invasive, forming fewer contacts with the vasculature and often displaying a more central position within the metastatic lesions (Fig. 4B,D–F; Movie 3). Long-term time-lapse analysis showed that, like primary tumours, invasive HT1080 cells could switch between cWnt^low^ and cWnt^high^ states within similar periods of time (Fig. 4G–J; Movie 3). Again, these switches resulted from changes in Venus fluorescence, as tdTomato fluorescence did not change significantly during the observation time (Fig. 4H,J). We noted there was an increase in the proportion of cWnt^high^ cells in HT1080 colonies, once they had formed (Fig. 4C), suggesting that canonical Wnt signalling becomes important for cell proliferation after invasion and colony establishment. However, we did not detect a sufficient number of cell division events *in vivo* to support this hypothesis.

Importantly, we obtained similar results when we switched fluorophores, using HT1080 7TCF-mCherry reporters in a stable GFP background (Fig. S3A,B). Within metastatic colonies, cWnt^high^ HT1080 7TCF-mCherry cells formed significantly shorter contacts with the vasculature and were significantly less invasive, ruling out artefacts coming from fluorescence imaging conditions used in these experiments (Fig. S3C,D). Moreover, similar results were observed using the human head and neck cancer cell line HEp3 engineered to express TOP-Venus and the non-responsive mutant FOP-Venus reporters with constitutive DsRed fluorescence. HEp3 cells form robustly growing metastatic lesions when injected i.v. into the chicken CAM vasculature (Stoletov et al., 2018), in a comparable manner to HT1080 cells. Within HEp3 metastatic colonies, cWnt^high^ cells formed significantly shorter contacts with the vasculature, compared to cWnt^low^ cells (Fig. S3E–G). No Venus signal was detected in the FOP-Venus HEp3 cells (Fig. S3F).

### Invasive HT1080 cells within primary tumours and metastatic lesions have high levels of ATF2-dependent pathway reporter activity

We wished to compare canonical and noncanonical Wnt signalling activities in HT1080 cells. The best known noncanonical Wnt reporter measures ATF2-dependent transcription in *Xenopus* embryos (Ohkawara and Niehrs, 2011). ATF2-dependent reporters have been used to measure Wnt signalling in other settings, but this remains a controversial area (see Introduction). Nevertheless, we wished to determine whether an ATF2-dependent reporter could be used as a readout for noncanonical Wnt signalling in HT1080 cells during cancer cell invasion *in vivo*, given that, to our knowledge, this has not been studied previously. To this end, we engineered HT1080 cells expressing an ATF2-dependent destabilised GFP reporter (Geetha-Loganathan et al., 2014) in the tdTomato background and characterised them *in vitro*. These cells display moderate activation of the reporter in a basal state and, upon exogenous expression of the prototypical noncanonical Wnt ligand Wnt-11, we could detect, by FACS, a shift to the right in the GFP signal, reflecting increases in the percentages of positive cells in both the total GFP and GFP^high^ populations (Fig. S4A,B). This increase was dependent on the amount of Wnt-11 plasmid transfected (Fig. S4C), partially inhibited by dominant-negative ATF2 (Fig. S4D) and reduced to a small extent by treatment with the Wnt secretion inhibitor LGK-974 (Fig. S4E) and to a greater extent by the JNK inhibitor JNK-IN-8 (Fig. S4F). ATF2-dependent transcription can also be activated in response to cell stress (Watson et al., 2017). To determine if the effect of Wnt-11 transfection on ATF2-GFP was cell stress dependent, experiments were carried out in the presence of the integrated stress response inhibitor (ISRIB) (Sidrauski et al., 2013). ISRIB significantly reduced activation of the ATF2-GFP reporter by tunicamycin, a known inducer of the ER stress response (Yang et al., 2020) (Fig. S4G). In contrast, ISRIB had no effect on the response to Wnt-11, suggesting that the effect of Wnt-11 does not involve induction of cell stress (Fig. S4H). These observations indicate that this reporter can respond to a non-canonical Wnt *in vitro*, although they do not rule out potential artefacts from expression of exogenous Wnt-11. Next, we examined reporter activity *in vivo*. As observed *in vitro*, the majority (>90%) of HT1080 cells in primary tumours displayed moderate ATF2-dependent GFP reporter activity (ATF2^pos^; green to red signal average ratio >1.5). There was a small proportion of cells with a low GFP/tdTomato fluorescence ratio (ATF2^low^ green to red signal average ratio <1.5) that was significantly less invasive and was predominantly located within the main tumour mass (Fig. S4I,J). Primary tumour cell-tracking analysis found that ATF2^low^ HT1080 cells invaded significantly more slowly and with less directionality than ATF2^pos^ cells (Fig. S4K,L).

**Fig. 5.**
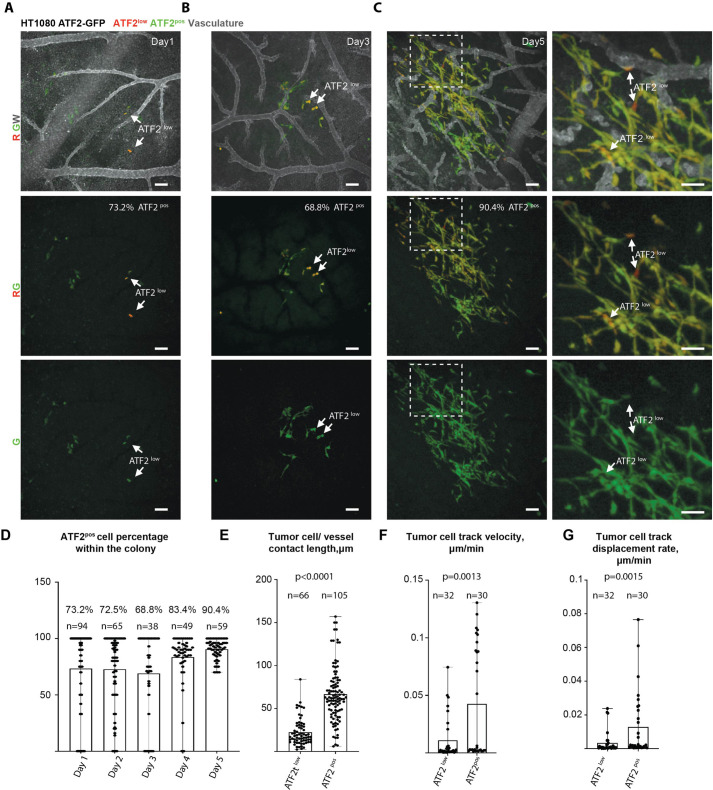
**ATF2 pathway reporter activity is upregulated during metastatic lesion invasion.** (A–C) Representative images showing ATF2 pathway reporter activity patterns within metastatic lesions formed by HT1080 cells 1–5 days post-injection. Examples of cancer cells that display low (ATF2^low^) ATF2 pathway reporter activity are highlighted by white arrows. Right-most panels for C show enlarged areas from within the dashed line squares. (D) Quantification of the percentage of ATF2^pos^ HT1080 cells in metastatic lesions 1–5 days post-tumour cell injection (dpi). (E) Quantification of the mean length of tumour cell–blood vessel contacts formed by ATF2^low^ or ATF2^pos^ HT1080 cancer cells within the metastatic cancer cell lesions at 5 dpi. (F) Quantification of mean ATF2^low^ and ATF2^pos^ cancer cell track velocity during the metastatic cancer cell lesion invasion. (G) Quantification of mean ATF2^low^ and ATF2^pos^ cancer cell track displacement rates during metastatic cancer cell lesion invasion. Statistical comparison was performed using a Mann–Whitney test. *n* animals=at least 11 for every time point in (D); 13 (E) and 5 (F,G). Scale bars: 50 µm.

A similar pattern of ATF2-dependent GFP reporter activity was observed in HT1080 metastatic lesions (Fig. 5A–C). Time-series analysis showed that, throughout the whole observation period, ATF2^pos^ HT1080 cells constituted the majority of the metastatic lesions and were predominantly located at the invasive edges of metastatic colonies, forming extended contacts with the CAM vasculature (Fig. 5D,E). In contrast, ATF2^low^ cells were located in the ‘central’ areas of the metastatic lesions and formed less extensive contacts with the vasculature (Fig. 5E). Tracking analysis showed that, similar to what was observed in primary tumours, ATF2^pos^ cells migrated significantly more quickly and with more directionality, compared to ATF2^low^ cells, when invading within the metastatic lesions (Fig. 5F,G). Importantly, HT1080 cells engineered to express GFP under the control of a mutated version of the ATF2-dependent promoter (ATF2Δ) displayed weak, patternless GFP expression throughout the metastatic lesions (Fig. S5A), consistent with the results observed using the wild-type ATF2-GFP reporter being dependent on ATF2 signalling. As expected, these cells did not respond to stimulation by Wnt-11 or to tunicamycin treatment (Fig. S5B,C).

### β-catenin/TCF-dependent and ATF2-dependent reporters display complementary activation patterns during cancer cell invasion within primary tumours and metastatic lesions

The finding that the β-catenin/TCF-dependent and ATF2-dependent pathway reporters showed nearly opposite activation patterns was intriguing and merited more detailed investigation. To this end, a double-reporter HT1080 cell line (HTDR) was engineered to expressed both ATF2-GFP and 7TCF-mCherry reporters, as well as constitutively expressing the cyan fluorescent protein mCerulean to normalise the fluorescence intensities of GFP and mCherry. FACS and immunofluorescence analysis confirmed HTDR cells responded to stimulation by recombinant Wnt-3a protein and Wnt-3a conditioned medium by activating 7TCF-mCherry, and to exogenous expression of Wnt-11 by activating ATF2-GFP, confirming the dual functionality of these reporter cells (Fig. S6A–D). Notably, HTDR cells also detected Wnt-11-induced inhibition of Wnt-3a activation of canonical signalling, as previously observed using luciferase-based gene reporter assays (Zhang et al., 2017), and there was a non-significant reduction of the Wnt-11 induced ATF2-GFP signal upon stimulation of cells with Wnt-3a-conditioned medium (Fig. S6B,C).

**Fig. 6.**
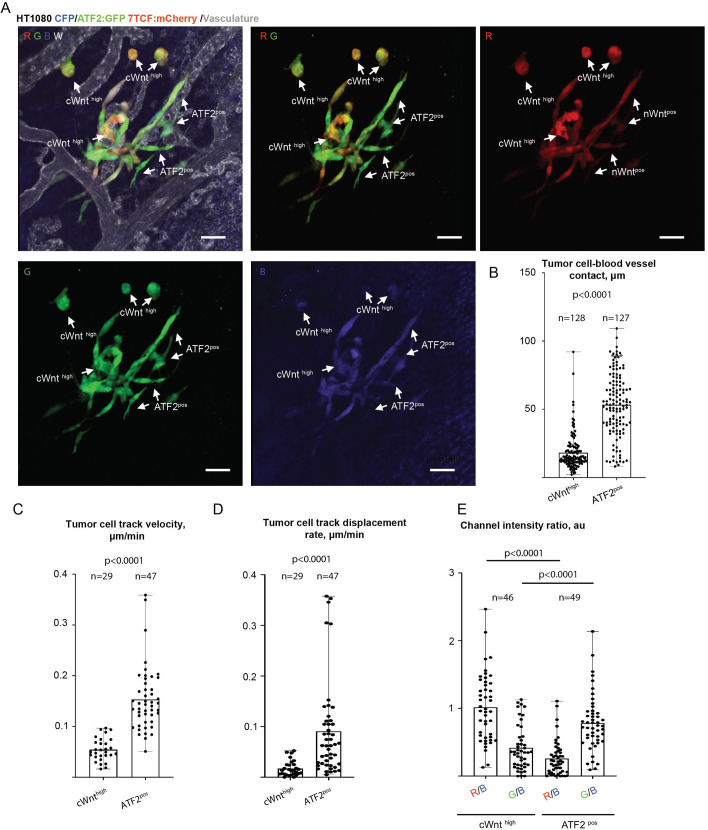
**Canonical Wnt and ATF2 pathway reporters show complementary activation patterns during metastatic lesion formation.** (A) Representative images showing Wnt canonical and ATF2 reporter activity patterns in the metastatic lesions formed by HTDR cells at 5 dpi. Separate panels show red/green (RG; mCherry/GFP), red (R), green (G) and blue (B; mCerulean) channels only. Examples of cWnt^high^ or ATF2^pos^ HTDR cells are highlighted by white arrows. (B) Quantification of the mean length of tumour cell–blood vessel contacts that are formed by cWnt^high^ or ATF2^pos^ HTDR cancer cells within the metastatic cancer cell lesions at 5 dpi. (C) Quantification of the mean cWnt^high^ or ATF2^pos^ HTDR cancer cell track velocity during the invasion of metastatic cancer cells. (D) Quantification of mean cWnt^high^ or ATF2^pos^ HTDR cancer cell track displacement rate during the invasion of metastatic cells. (E) Quantification of red/blue or green/blue (mCherry/mCerulean or GFP/mCerulean) channel intensity ratios for individual cWnt^high^ or ATF2^pos^ HTDR cells (see also Movie 4). Results in B–E are mean with error bars showing the complete range of results. Statistical comparison was performed using Mann–Whitney test. *n* animals=9 (B); 5 (C,D) and 8 (E). Scale bars: 50 µm.

We first visualised metastatic lesions formed by HTDR cells (Fig. 6A). Importantly, we observed roughly similar patterns of 7TCF-mCherry and ATF2-GFP reporter expression, as previously observed in HT1080 cells expressing the individual reporters. cWnt^high^ HTDR cells (red to green signal average ratio >1.5) were more rounded and located within the central regions of metastatic lesions, where cells are normally closely packed (Fig. 6A; Fig. S6E). Additionally, cWnt^high^ cells formed significantly fewer extended contacts with the CAM vasculature, compared to ATF2^pos^ cells, and were less migratory (Fig. 6B,D). Long-term time-lapse analysis of metastatic lesions formed by HTDR cells provided striking confirmation of the results obtained from the single-reporter cell experiments (Movie 4). Indeed, cWnt^high^ HTDR cells did not actively participate in invasion, in clear contrast to ATF2^pos^ HTDR cells, which rapidly and persistently invaded out of metastatic lesions, as confirmed by tracking analysis (Fig. 6C,D). The apparent complementary pattern of 7TCF-mCherry and ATF2-GFP activities was further confirmed by direct measurement of the fluorescence intensity within individual HTDR cells normalised to mCerulean intensity (Fig. 6E). On average, HT1080 cells with higher 7TCF-mCherry fluorescent signal intensity had lower ATF2-GFP signal intensity (Fig. 6E).

**Fig. 7.**
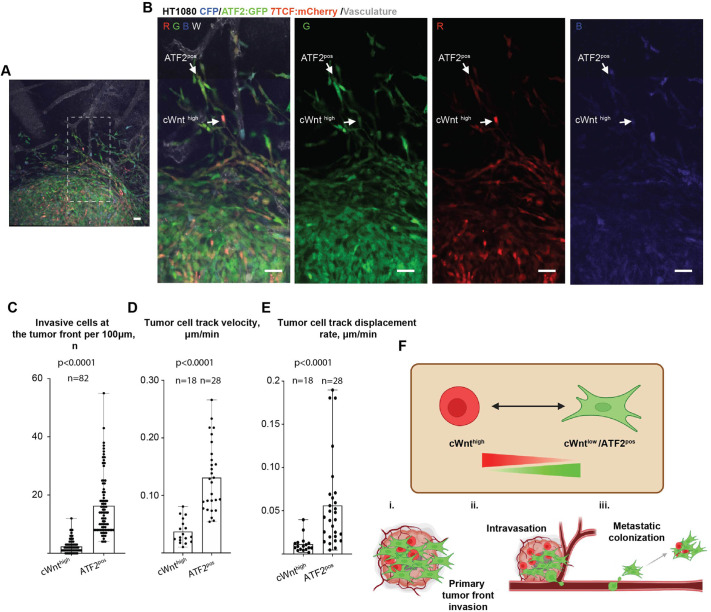
**Canonical Wnt and ATF2 pathway reporters show complementary activation patterns during primary tumour invasion.** (A) Representative images showing Wnt canonical and ATF2 activity patterns within the HTDR cancer cell induced primary tumours at 5 dpi. (B) Separate panels show red/green (RG; mCherry/GFP), red (R), green (G) and blue (B; mCerulean) channels only. Examples of cWnt^high^ or ATF2^pos^ HTDR cells are highlighted by white arrows. (C) Quantification of cWnt^high^ or ATF2^pos^ HTDR cells at the primary tumour invasive front 5 days post-tumour cell inoculation. (D) Quantification of mean cWnt^high^ or ATF2^pos^ HTDR cancer cell track velocity during invasion. (E) Quantification of mean cWnt^high^ or ATF2^pos^ HTDR cancer cell track displacement rate during invasion (also see Movie 5). (F) Proposed model for the complementary activation of canonical (cWnt) and ATF2 signalling and their roles in primary tumour front invasion (i), intravasation (ii) and metastatic colonisation (iii). Statistical comparison was performed using a Mann–Whitney test. *n* animals=12 (C) and 5 (D,E). Scale bars: 50 µm.

Live imaging of primary tumours formed by HTDR cells further confirmed the activation patterns observed in the metastatic lesions during cancer cell invasion, with ATF2^pos^ cells displaying higher invasive capacity, in contrast to cWnt^high^ cells (Fig. 7A,B; Movie 5). Even though cWnt^high^ HTDR cells constituted a significant part of the main tumour mass (19.7±9.6%; mean±s.d.), they were significantly underrepresented within the invasive tumour front, displaying slower and less directional invasion. In contrast, ATF2^pos^ HTDR cells rapidly invaded, both at the primary tumour front and within the main tumour mass, confirming the data obtained using cells expressing single-pathway reporters (Fig. 7C–E; Movie 5).

## DISCUSSION

We have visualised the activation patterns of β-catenin/TCF-dependent and ATF2-dependent reporters during human cancer cell invasion and metastasis. To our knowledge, this work is the first to simultaneously image these reporter activities *in vivo* in real-time within the same invading cancer cells. Canonical Wnt reporter activity was observed to be significantly downregulated during cancer cell invasion, both in primary tumours and in metastatic lesions. Cells displaying increased canonical Wnt reporter activity (cWnt^high^) tended to remain within the primary tumour or in non-invasive zones of metastatic lesions. This was found to be the case in multiple cancer types – fibrosarcoma (HT1080), head and neck cancer (HEp3) and glioblastoma (U87, U118) – and was observed using both Venus and mCherry as reporting fluorophores for β-catenin and TCF-dependent activation. Even though cWnt^high^ cells were often found to be in close association with the primary tumour vasculature, they formed less extensive contacts and did not participate in intravasation. Time-series analysis found that the vast majority of metastatic lesions consisted of cWnt^low^ cells during the initial stage of metastatic lesion formation, further suggesting that cWnt^low^ cells are responsible for the initial steps of the metastatic cascade (invasion, intravasation and extravasation). Importantly, we showed that invading cancer cells rapidly (within less than 24 h) undergo shifts between cWnt^high^ and cWnt^low^ states, both in primary tumours and metastatic lesions. In clear contrast, invading cancer cells maintained relatively high levels of ATF2-dependent reporter activity (ATF2^pos^), both within the metastatic lesions and in primary tumours. Moreover, time-series analysis of metastatic lesions found that the majority of metastatic cells were ATF2^pos^ and that the ATF2^low^ cells were located in the central areas of metastatic lesions and displayed non-invasive behaviour. Simultaneous visualisation of both reporters confirmed that they display distinct and highly coordinated activation patterns during metastatic cancer progression.

Based on these results, we hypothesise that, during the initial steps of the metastatic cascade (invasion, intravasation, extravasation), low Wnt canonical activity is a pre-requisite for invasion and intravasation. In contrast, high ATF2-dependent signalling promotes cancer cell invasion out of the primary tumour and dissemination to secondary sites of metastasis. Shortly after extravasation at a secondary site, canonical Wnt activity is upregulated, which could facilitate the proliferation of the metastatic lesion, whereas ATF2-dependent activity remains high in cells invading out of the metastatic lesions, leading to the invasive expansion of the distant metastatic lesion. These observations are summarised in the model shown in Fig. 7F. In the primary tumour, canonical Wnt signalling is mainly limited to the main tumour mass, where it might be responsible for driving cancer cell proliferation (Fig. 7Fi). β-catenin/TCF-dependent signalling is downregulated during primary tumour invasion (Fig. 7Fi) and intravasation (Fig. 7Fii), while a sufficient level of ATF2-dependent activity is maintained. Shortly after extravasation, canonical Wnt activity is upregulated again, likely driving metastatic lesion proliferation (Fig. 7Fiii). Indeed, during our intravital imaging experiments, we detected that increased β-catenin/TCF-dependent signalling often preceded cell division events, both within the primary tumour and in metastatic lesions. However, the low frequency of these events did not allow us to draw any quantitative conclusions (Figs 1G and 4G). Similarly, the cancer cells that are responsible for metastatic lesion invasion show reduced β-catenin/TCF-dependent signalling, while maintaining sufficient levels of ATF2-dependent activity (Fig. 7Fiii).

We provide another observation of a highly coordinated pattern of β-catenin/TCF-dependent and ATF2-dependent pathway activation. This was hinted at by the opposite behaviours observed by the single-reporter cell lines and was further confirmed using the double-reporter cell line (HTDR). The dual reporter cell line gave us striking confirmation of the findings observed using single reporters, yet these reporters are different. For example, these reporters showed differences in the percentages of the cWnt^high^ tumour cells within the main tumour mass, as quantified for single HTTOP or dual HTDR cancer cell lines, which can be explained by the cells being selected for optimal reporter responses to different signals during FACS sorting for cell line generation. Although crosstalk between canonical and noncanonical Wnt signalling has been reported previously (Zhang et al., 2017; Watson et al., 2017; Ma et al., 2022), this is the first time β-catenin/TCF-dependent and ATF2-dependent branches of signalling have been monitored simultaneously *in vivo* during cancer cell metastasis. Moreover, a similar complementary behaviour of Wnt and ATF2-dependent reporters has been reported in the developing rat brain (Boitard et al., 2015). Many canonical Wnt signalling target genes are negative-feedback regulators, including noncanonical Wnt pathway components (DKK1, WNT11 and ROR proteins; Niida et al., 2004; Dwyer et al., 2010; Yamagata et al., 2012). These targets can both repress canonical signals and induce the activation of noncanonical signals. In fact, many of the reports that point to β-catenin-dependent invasion might be detecting downstream effects on noncanonical Wnt signalling. Indeed, invasive behaviour of breast cancer cells is inhibited if β-catenin signalling is disrupted (Dwyer et al., 2010). These complementary patterns were observed also *in vitro*, where activation of 7TCF-mCherry was repressed by exogenous expression of Wnt-11 (Fig. S4A–C). Although ATF2-dependent reporters have been used as a readout for noncanonical Wnt signalling in many studies (Ohkawara and Niehrs, 2011; Yamamoto et al., 2008; Murillo-Garzón et al., 2018; Geetha-Loganathan et al., 2014; Sidrauski et al., 2013; Gao et al., 2020) their use in mammalian cells remains controversial. Of relevance to our study, the same construct used here to monitor ATF2-dependent activity has been reported to function as a noncanonical reporter in rat embryos (Boitard et al., 2015) and furthermore, it was found to behave with a similar complementary pattern to a Wnt canonical reporter. Noncanonical Wnt signals are better known for cytoskeletal remodelling and their effects on transcription are less direct. Moreover, ATF2-dependent signalling is only partially representative of a range of transcription factors that can be regulated by noncanonical Wnt signals (Wallkamm et al., 2014). Conversely, Wnt-independent signals, such as ER stress, also lead to activation of ATF2-dependent transcription (Yamagata et al., 2012). The mechanism of activation of this reporter by ectopically expressed Wnt-11 *in vitro* does not appear to involve cell stress signalling, as it was not inhibited by ISRIB, which blocked reporter activation by tunicamycin, an inducer of ER stress (Fig. S4E–H). Activation was reduced by JNK inhibition and by a dominant-negative form of ATF-2 that forms inactive dimers with ATF2 and its partners, but was only moderately (10–20%) inhibited by LGK974, suggesting it is not solely driven by secreted Wnt-11. Thus, the mechanism of activation by exogenous Wnt-11 remains to be determined. The moderately high basal ATF2 reporter activity observed in cells *in vivo* might result from the convergence of multiple signals on ATF2-dependent signalling and the extent to which noncanonical Wnt signals play a role remain to be determined. The *in vivo* studies nevertheless highlight the potential importance of ATF2-dependent signalling for cancer cell invasion and metastasis. Overall, our results have several implications. First, they argue that conditions that favour low canonical Wnt signalling correlate with increased cell motility. Indeed, according to our model, downregulation of Wnt/β-catenin activity alone could lead to enhanced noncanonical activities, including ATF2-dependent activity (Fig. 7F), and increased cancer cell invasion and intravasation. This could be particularly important for rapidly invading cancers, such as glioblastoma. We believe that this level of complex signalling driving distinct cell invasive phenotypes means that a targeted approach to Wnt inhibition is needed to avoid unwanted effects of inhibiting beneficial Wnt signals, for example, by identifying specific vulnerabilities at the level of Wnt co-receptor interactions (van Amerongen, 2012; Eubelen et al., 2018).

Several questions remain to be investigated. Are cWnt^high^ cells truly responsible for driving cancer cell proliferation within the primary tumour and metastatic lesions? Can any cancer cell switch into the cWnt^high^ state, or is this ability limited to a CSC-like population? How specific are the β-catenin and TCF- and ATF2-dependent reporters *in vivo*, which Wnt ligands are responsible for their activation and what is their source? Finally, despite a long history of use in the field of developmental and cancer biology, questions remain on how closely the results obtained in the chick CAM model, as a xenograft host, can be translated into human cancer settings. For instance, the embryonic environment could be providing a rich source of chick Wnt ligands that share high sequence identity with human Wnts and are therefore likely to activate the reporter cells monitored here. This might also be the case in mouse xenograft studies, although Wnt-dependent activity in adults may be more niche-restricted than in embryos (Steinhart and Angers, 2018). The difficulty of continuous, high-resolution intravital imaging in more similar animal systems is currently precluding an answer to this question, and therefore other, non-imaging-based approaches might be required to address this.

## MATERIALS AND METHODS

### Cells and reagents

The human fibrosarcoma cell line HT1080, epidermoid carcinoma cell line HEp3 and human glioblastoma cell lines U87 and U118 were all maintained as described previously (Stoletov et al., 2018; Latour et al., 2021) pBARV (TOP-Venus) and control fuBARV (FOP-Venus) vectors, kindly provided by Jeanot Muster and Randall Moon (University of Washington, Seattle, USA; Biechele and Moon, 2008) were transduced to the parental HEp3 cell line, and to HT1080 cells constitutively expressing tdTomato (HT1080-tdTomato described previously; Leong et al., 2014) using the following lentiviral transduction protocol. Briefly, HEK293FT packaging cells (Thermo Fisher Scientific) were transfected using calcium phosphate with a third-generation plasmid system (pREE, pRSV-Rev and VSV-G) together with the TOP and FOP-Venus vectors alone (HT1080-tdTomato) or with TOP- and FOP-Venus that had additional stable dsRed expression vectors (HEp3). Target cells were transduced for two consecutive days with lentiviral particles contained in the HEK293 cell conditioned medium. At 5 days post infection, cells were stimulated with Wnt-3a conditioned medium and cells expressing Venus/dsRed were FACS sorted, after which a counter-selection of negative cells under non-stimulated conditions was performed, as described previously (Leong et al., 2014). The glioblastoma U87 and U118 cell lines were kindly provided by Dr Mary Hitt laboratory (University of Alberta, Edmonton, Canada). Cells were stably transduced with 7xTCF-mCherry (Addgene #24315) and pLVE-eGFP (Addgene #52581) using HEK293 FT Thermo Fisher ViraPower^TM^ lentiviral packaging mix transfection protocol, as described by the manufacturer. HT1080-ATF2-GFP cells were generated as described above transducing HT1080-tdTomato with ATF2-GFP vector [pCLX CHOP-dGFP (Shahcheraghi et al., 2020); Addgene #71299, kindly deposited by Patrick Salmon, University of Geneva]. ATF2-GFP transduced cells were FACS-sorted upon stimulation by amino acid starvation for 16 h, which has been described previously to activate this reporter (Bruhat et al., 2000). All the cell lines were tested for contamination. The ATF2 mutant GFP reporter (ATF2Δ-GFP) was generated by AvaI digestion and re-ligation of pCLX-CHOP-dGFP (Addgene #71299) to remove the region containing the ATF2-binding site. The mutated reporter was transduced as for the wild-type reporter and a control GFP reporter driven by a constitutive UbC promoter (Addgene #27245). Cells expressing the mutated reporter were GFP low or negative, as assessed by FACS, and non-responsive to ATF2-activating stimuli. To verify the presence of the reporter in the cells, genomic DNA was analysed by qRT-PCR using primers (sequences available upon request) for GFP and for tdTomato (constitutively expressed at similar levels in all the reporter cell lines), which showed that the mutated ATF2 reporter plasmid was incorporated at a copy number in the same range as those for the GFP control and wild-type ATF2-GFP reporters. The double-reporter cell line HTDR was generated by transfecting 7xTCF-mCherry (Addgene #24315) and pCLX-CHOP-dGFP into HT1080 cells that had been generated to stably express mCerulean (pRBOW vector, kindly provided by Dr Andries Zijlstra, Vanderbilt University, Nashville, USA) using Thermo Fisher ViraPower^TM^. Conditioned media were collected from L-Wnt-3a and control LM-TK- cells, following the ATCC protocol. CM were filtered through 0.22 μm Millex-GP filters (Merck-Millipore) and was used in a 1:1 ratio with fresh medium for the stimulation. DnATF2 (CMV500 A-ATF2) and empty vector (CMV500) plasmids were Addgene plasmid #33353 (deposited by Charles Vinson) and were transiently transduced to cells using LTX/Plus reagents following the manufacturer's instructions. Human recombinant Wnt-3a protein was purchased from R&D systems, Bio-Techne (Wnt-3a: 5036-WN-010) and was used at 100 ng/ml. The GSK-3 inhibitor CHIR99021 was purchased from Selleck (Cat. # S1263) and used at 5 μM. The porcupine inhibitor LGK974 was from Selleck (Cat. # S7143). JNK-IN-8 inhibitor was from Sigma-Aldrich (cat. #SML1246) and used at 10 μM. Integrated Stress Response inhibitor (ISRIB) was from Sigma-Aldrich (cat. #SML0843) and was used at 200 nM. Tunicamycin was from Sigma-Aldrich (cat. #T7765) and was used at 2 μg/ml.

### t-scratch *in vitro* migration assays

HT1080-TOP-Venus cells were plated in six-well plates at high-density. Cells were pre-treated overnight with the canonical Wnt pathway activators: CHIR99021 (5 μM) and Wnt-3a recombinant protein (100 ng/ml) in DMEM containing 2% FBS. The next day a confluent cell monolayer was scratched twice per well using a 200 μl pipette tip and media were subsequently renewed with DMEM containing 2% FBS containing and treatments. Wounds were imaged at time 0 h in six predetermined marked locations and then allowed to migrate to close the wound for 5–8 h, when the scratches were imaged again in the same locations. Scratch closure was determined using ImageJ plugin *MRI Wound Healing Tool* (https://github.com/MontpellierRessourcesImagerie/imagej_macros_and_scripts/wiki/). Area measurements at endpoint were made relative to the same area at time 0 h (*x*) and re-calculated relative to closure (1−*x*). Relative closure data are from three independent experiments and are presented relative to the control condition.

### Fluorescence activated cell sorting

Reporter cells were characterised and sorted using a FACSAria (Becton Dickinson) flow cytometer, using the non-transduced and/or unstimulated cells as controls to define gating and the data were analysed using the FACSDiva software. Representative FACS plots images are presented, and quantitative data correspond to three independent experiments.

### *Ex ovo* chick embryo cancer xenograft model

Fertilised White Leghorn chicken eggs were obtained from University of Alberta Poultry Research Centre and maintained in a humidified incubator at 38°C. All animal experiments were performed according to approved guidelines. After 4 days of incubation, embryos were removed from their shells using a Dremel tool with a cutting wheel and maintained under shell-less conditions, in a covered dish in a humidified air incubator at 38°C and 60% humidity as previously described (Stoletov et al., 2018; Leong et al., 2014).

For the primary tumour imaging, day 10 chicken embryos were injected with 10^5^ (HT1080, U118 or U87) cells in PBS directly in between CAM ectoderm and endoderm layers. Sterilised, rounded (22 mm) coverslips were applied on top of the tumour. At 24 h post tumour cell application and invasive fronts were imaged 5 days post tumour cell inoculation.

For metastatic colony imaging, day 10 chicken embryos were injected intravenously with 2.5×10^4^ HT1080 or HEp3 cells. Sterilised, rounded (22 mm) coverslips were applied on top of the CAM containing metastatic colonies at 24 h post cancer cell injection. Metastatic colonies were allowed to grow for 1–5 days, and individual, non-overlapping metastatic colonies were selected for imaging. Chicken CAM vasculature was visualised using Lectin-649 (DyLight, Vector Laboratories, cat. DL-1048) injection, as described previously (Stoletov et al., 2018; Leong et al., 2014).

### *In ovo* chick embryo spontaneous metastasis model

Briefly, the *in ovo* chick metastasis model was used as described before (Palmer et al., 2011). Fertilised White Leghorn chicken eggs were maintained in a humidified incubator at 38°C. After 10 days of incubation, the chick CAM was dropped and a small 1 cm^2^ window was cut in the eggshell. HT1080 (2×10^5^) cells were applied onto the CAM surface and the eggshell was sealed using sterile tape, and eggs were incubated for 5–7 days more. On days 5 or 7 post-tumour cell inoculation, Lectin-649 was injected intravascularly to label the vasculature and the lower CAM was separated. Lower CAM metastatic colonies were imaged using a Nikon-A1 confocal microscope (10×) and the number of metastatic cancer cells per 10× field (5 animals per condition) was quantified.

### Image acquisition and analysis

Real-time imaging of cancer cell invasion was performed by acquiring 4-dimensional image series of single cancer cells within the CAM tissue. A 50–300 µm image stack was acquired every 10–20 min in 2–10 µm step size increments for 8–48 h. A Nikon A1r upright microscope (Nikon) fitted with a temperature regulated enclosure and a range of Nikon microscope objectives [10×, 25× (WI)] was used for multicolour imaging of cancer cells and chicken embryo CAM vasculature. Image drift was corrected using the ImageJ StackReg plugin (Biomedical Imaging Group; http://bigwww.epfl.ch/) prior to tracking analysis. Time 0 was defined as the time of the first image capture. Individual cells were tracked using a built in Volocity Object tracking module. Track velocity was calculated as an average speed of the track. Track displacement rate (productivity) was calculated using the built in Volocity module as total track displacement (straight line distance from the first track position to the last) divided by track time. For quantification of the cancer cell number per colony and invasive cancer cells at the tumour front, 25× *z*-stack images were acquired (2–5 µm step) and cancer cells were counted manually using Nikon Elements software. *Z*-stack maximum intensity projections that contain all optical slices were used for quantification of the invasive cancer cells at the tumour front. For quantification of the cancer cell–blood vessel contact length, 25× *z*-stack images were acquired (2–5 µm step) and cancer cell–blood vessel contact length (maximum span) was measured using Nikon Elements software built in length measurement module. For quantification of tumour cell intravasation, individual primary tumour *z*-slices (25×, 1–2 µm step) were analysed using ImageJ software and intravascular tumour cell area was measured. *Z*-slices that contained the maximum diameter of blood vessels where tumour cell intravasation was detected were used for analysis. ImageJ built-in particle analysis (circularity) was used for cancer cell shape analysis. 25 individual optical slices from independent tumours (one slice/tumour) were used to quantify the percentage of cWnt^high^ cells for HT-TOP and HTDR cancer cell lines. For immunofluorescence, images were acquired using the 25× objective, 1–2 µm step. Cell percentage quantifications were performed based on ten independent fields and experiments were repeated at least three times.

All experimental data were plotted (mean with range plots, GraphPad Prism) and analysed for statistical significance using GraphPad Prism analysis module (unpaired two-tailed *t*-test or Mann–Whitney test).

## Supplementary Material

10.1242/joces.260285_sup1 Supplementary information
