# Supplementary material for: Intravital imaging of Wnt/β-catenin and ATF2-dependent signalling pathways during tumour cell invasion and metastasis
Source: J Cell Sci. 2023 Feb 10;136(3):jcs260285. doi: 10.1242/jcs.260285 (PMC10022745; doi:10.1242/jcs.260285)
Supplement: Supplementary information [file joces-136-260285-s1.pdf]

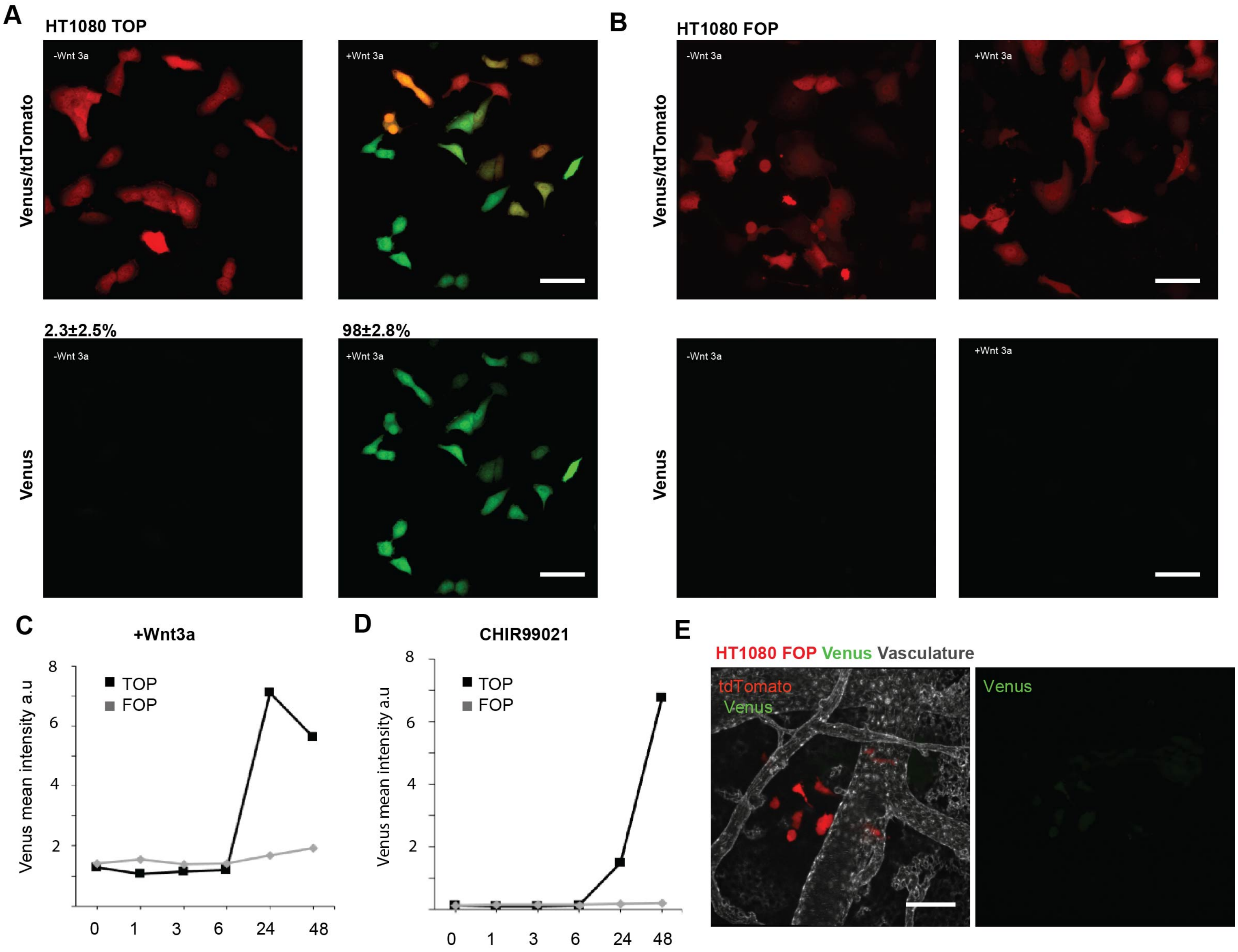

Supplementary Figure 1

Fig. S1. Characterisation of canonical Wnt reporter cancer cells used in the experiments. (**A-B**) Representative images showing responses of HT1080 TOP Venus (**A**) and HT1080 FOP Venus (**B**) cancer cells to Wnt-3a stimulation (100 ng/ml). **C-D**) FACS analysis showing stimulation with canonical activators of HT1080 TOP-Venus and HT1080 FOP-Venus cells. Venus fluorescence mean intensity time course upon stimulation with Wnt-3a-conditioned media (**C**) and CHIR99021 (**D**; 5 μM). **E**) Representative images showing HT1080 FOP Venus metastatic colony (5 dpi). Note that no Venus fluorescence is detected (right panel). Scale bars = 50 μm.

Journal of Cell Science • Supplementary information

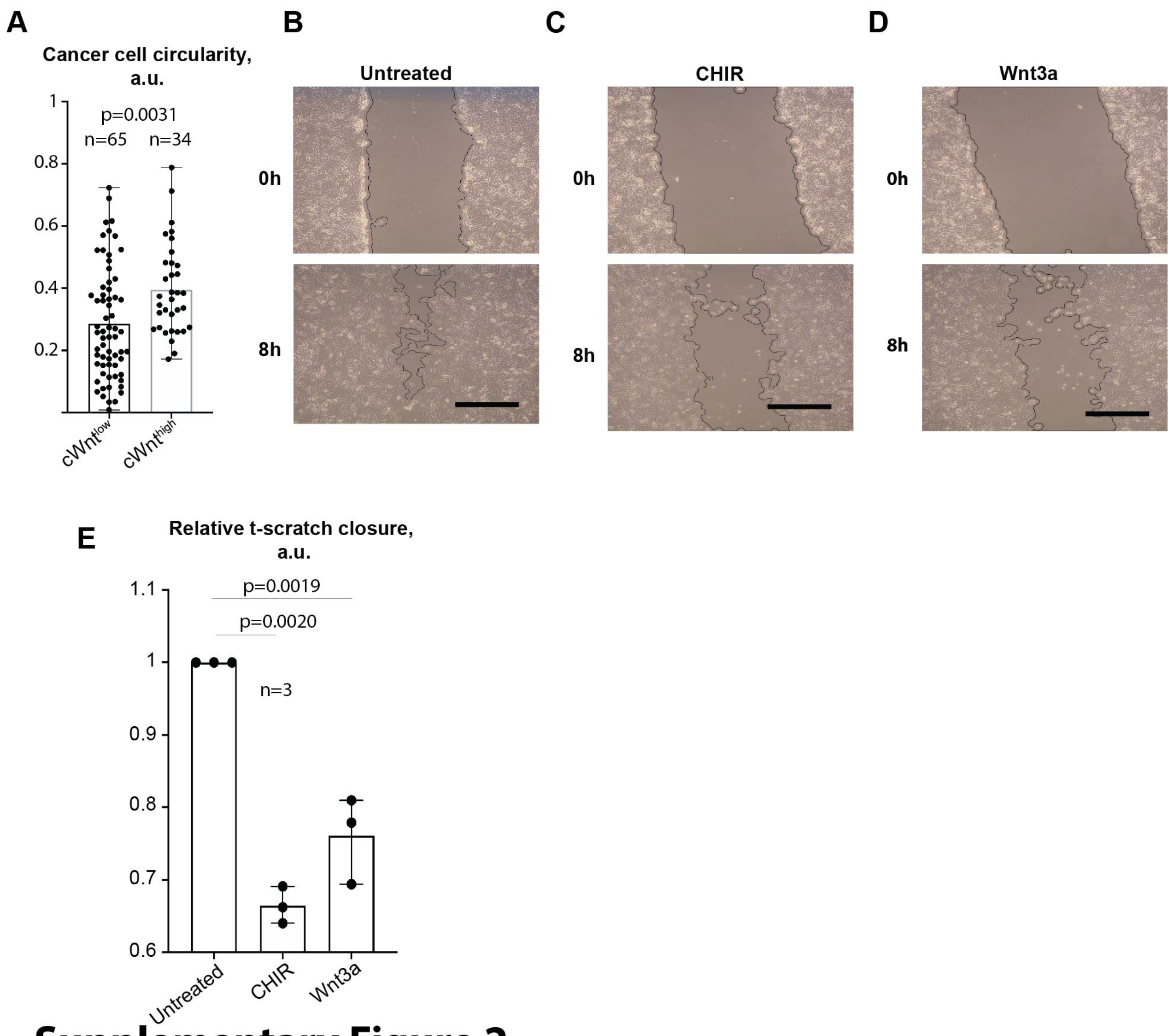

Supplementary Figure 2

Fig. S2. Cell shape (*in vivo*) and migration differences (*in vitro*) between cWnt^{high} and cWnt^{low} cells. (**A**) Cell shape (circularity) differences between cWnt^{high} and cWnt^{low} cells. (**B-C**) Representative image showing t-scratch at 0 and 8 h of HT1080-TOP-Venus cells untreated (**B**), treated with 5 μM CHIR99021 (**C**) and treated with 100 ng/ml of recombinant Wnt-3a protein (**D**). **E**) Quantification of relative t-scratch closure representing migration of HT1080 cells upon treatment with the aforementioned canonical Wnt pathway activators. Statistical comparison was done using unpaired t-test. n animals = 7 (**A**). Scale bars = 300 μm.

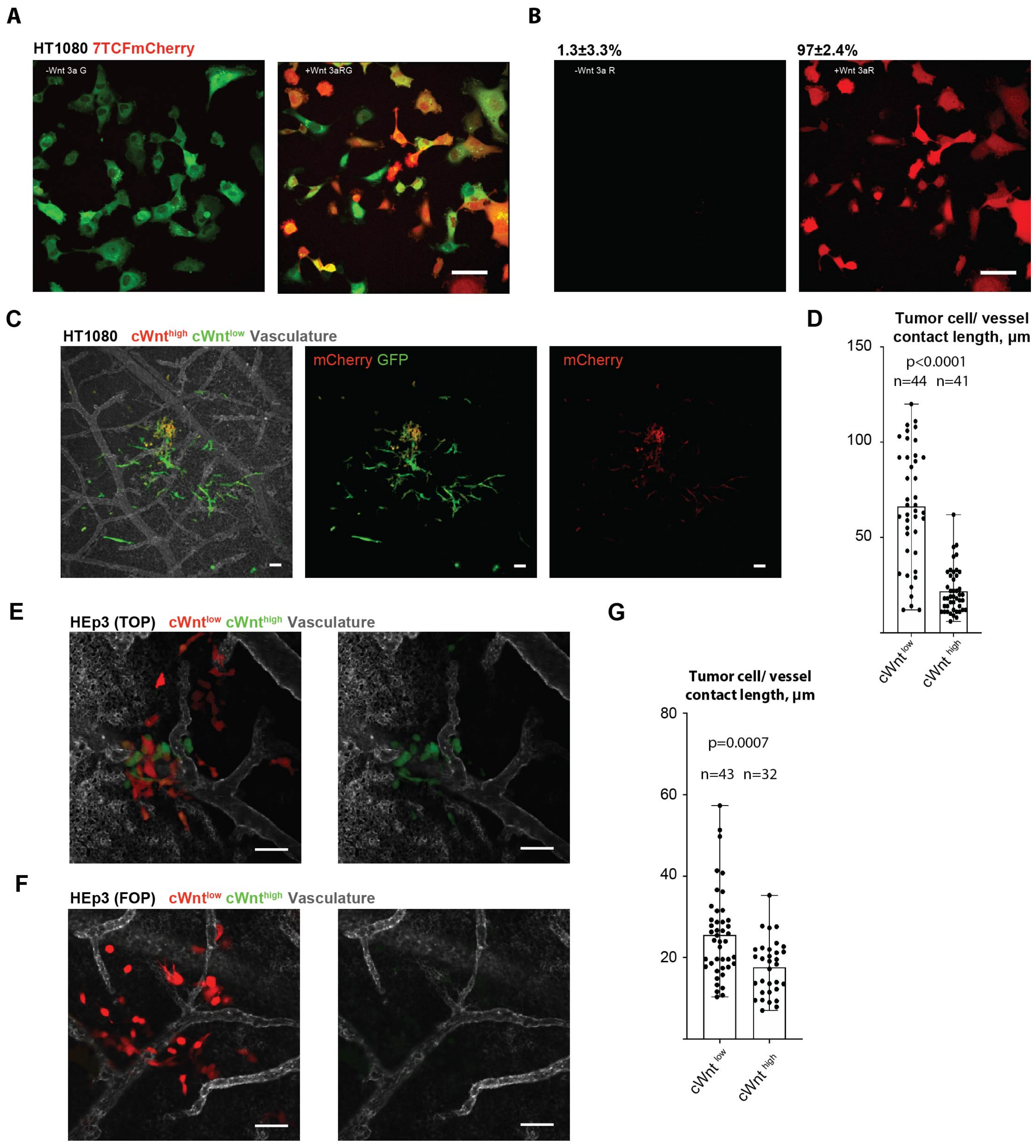

Supplementary Figure 3

Fig. S3. Characterization of HT1080 7TCFmCherry /GFP and Hep3 TOP/FOP-Venus/DsRed reporter cancer cells. (**A-B**) Representative images showing the response of HT1080 7TCF mCherry cells to Wnt-3a stimulation (100 ng/ml). **C**) Representative images showing a HT1080 7TCFmCherry metastatic colony (5 dpi). Middle and right-most panels show mCherry/GFP or mCherry channels only. **D**) Quantification of average tumour cell blood vessel contacts established by HT1080 7TCF mCherry cells. **E-F**) Representative images showing HEp3 TOP and FOP tdTomato/Venus metastatic colonies (5dpi). Note that no Venus fluorescence is detected in FOP cells. **G**) Quantification of average tumour cell blood vessel contacts established by HEp3 TOP and FOP tdTomato/Venus cells. Statistical comparison was done Mann-Whitney test. n animals = 7 (**D**) and 6 (**G**). Scale bars = 50 µm.

Journal of Cell Science • Supplementary information

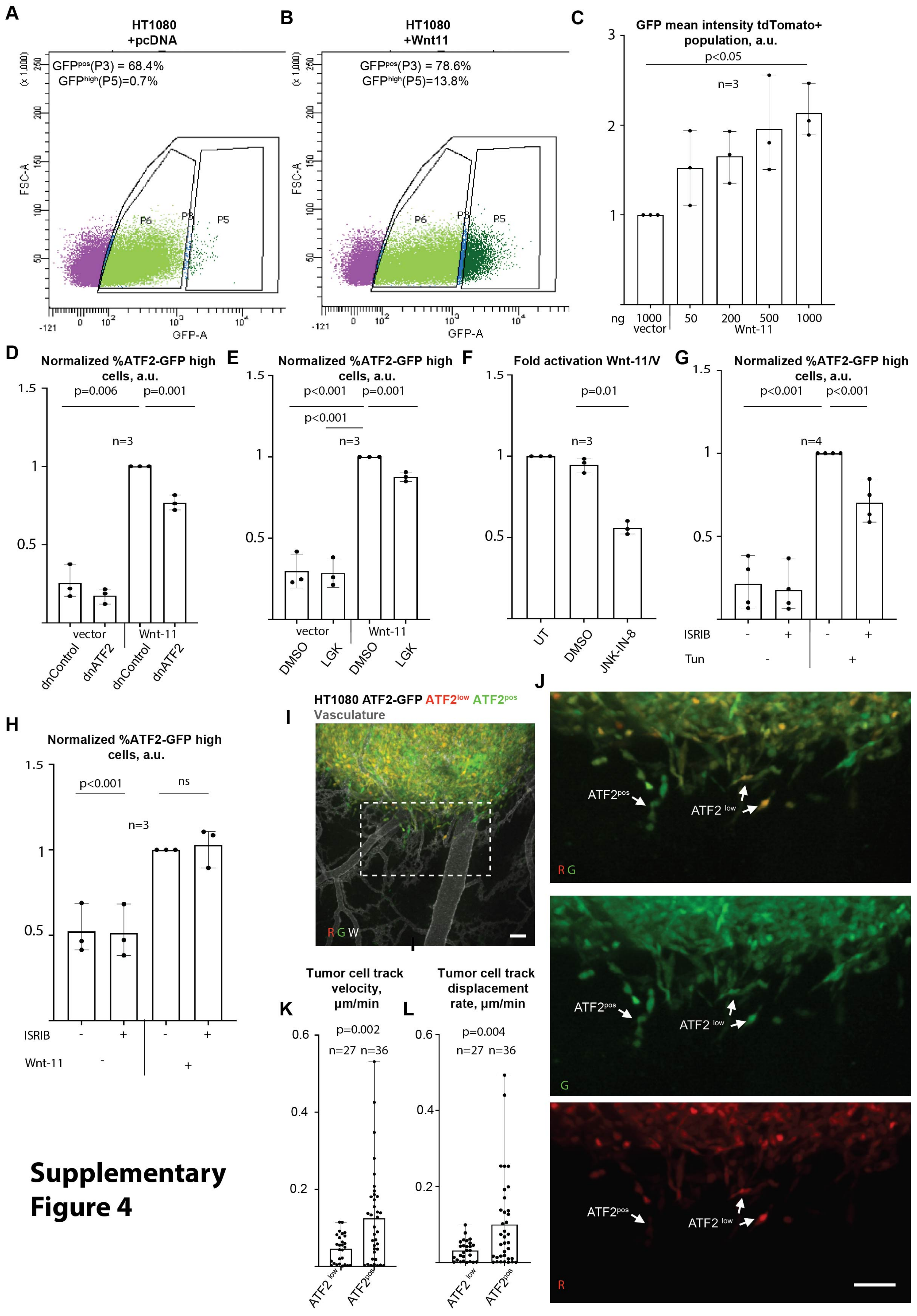

Supplementary Figure 4

Journal of Cell Science • Supplementary information

Fig. S4. **ATF2 pathway reporter activity is upregulated during primary tumour invasion.** (**A**-**B**) Representative FACS plots showing activation of ATF2 driven GFP expression in HT1080 cells transfected with empty vector (pcDNA) (**A**) and Wnt-11 (**B**), showing percentages of cell populations gated for total GFP, GFP^{pos} (P3), moderate GFP (P6) and GFP^{high} (P5). **C**) Plot showing activation of ATF2-GFP in HT1080 cells upon transfection of empty vector (pcDNA, vector) and increasing amounts of Wnt-11 plasmid (ng per well in a 6-well plate). **D**) Plot showing repression of Wnt-11-mediated activation of ATF2-GFP in HT1080 cells expressing empty vector (control, CMV-500) or dominant-negative ATF2 (dnATF2). **E**) Plot showing activation of ATF2-GFP in HT1080 cells upon expression of empty vector pcDNA (vector) or Wnt-11 in the presence of the porcupine inhibitor LGK-974 (1 mM; 16 h) or vehicle (DMSO). **F**) Plot showing the fold-change activation of the ATF2-GFP signal in HT1080 cells upon expression of Wnt-11 or empty vector pcDNA (Wnt11/V), in the presence of the JNK inhibitor JNK-IN-8 (10 mM; 16 h), vehicle (DMSO) or untreated (UT). **G**) Plot showing activation of ATF2-GFP in HT1080 cells treated with tunicamycin (2 mg/ml; 16 h) with or without ISRIB (200 nM; 16 h). **H**) Plot showing activation of ATF2-GFP in HT1080 cells upon expression of empty vector pcDNA (-) or Wnt-11 (+) with or without ISRIB (200 nM; 16 h). **I**) Representative image showing overall pattern of noncanonical pathway Wnt activity during the invasion of HT1080 cells in primary tumours. **J**) Images show red/green (tdTomato/GFP); green only and red only channels for the invasive front area from within the dashed square in (**I**). Examples of cancer cells that display high ($ATF2^{pos}$) or low ($ATF2^{low}$) ATF2 pathway activity are highlighted by white arrowheads. Chicken vasculature is stained with Lectin- 647 (white, W). **K**) Quantification of average $ATF2^{low}$ and $ATF2^{pos}$ cancer cell track velocity. **L**) Quantification of average $ATF2^{low}$ and $ATF2^{pos}$ cancer cell track displacement rate. Statistical comparison (unpaired t-test (**C-H**) or Mann-Whitney test (**K**, **L**)). n animals = 6 (**K**, **L**). Scale bars = 50 µm.

Journal of Cell Science • Supplementary information

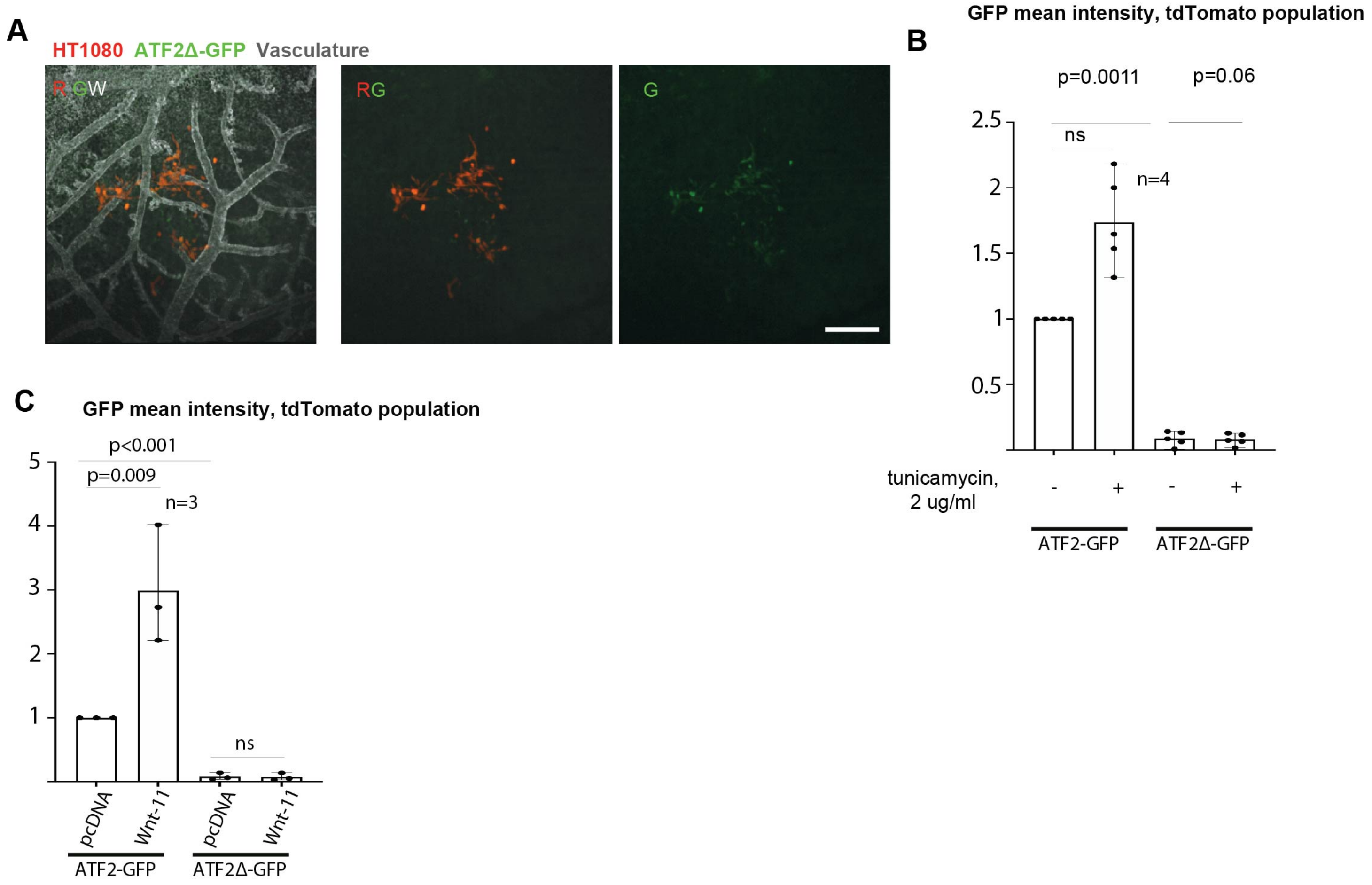

Supplementary Figure 5

Fig. S5. Characterization of HT1080 ATF2D-GFP (mutated ATF2 binding site) reporter cancer cells. (A) Representative images showing metastatic lesions formed by HT1080 cells engineered to express ATF2D-GFP, 5 days post-injection. **B)** Plot showing activation of ATF2-GFP and mutated ATF2D-GFP in HT1080 cells ER-stress inducer tunicamycin (2 µg/ml; 16 h). **C**) Plot showing activation of ATF2-GFP and the mutated reporter ATF2Δ-GFP in HT1080 cells upon expression of empty vector pcDNA or Wnt-11. Statistical comparison was done unpaired t-test. Scale bars = 50 µm.

Journal of Cell Science • Supplementary information

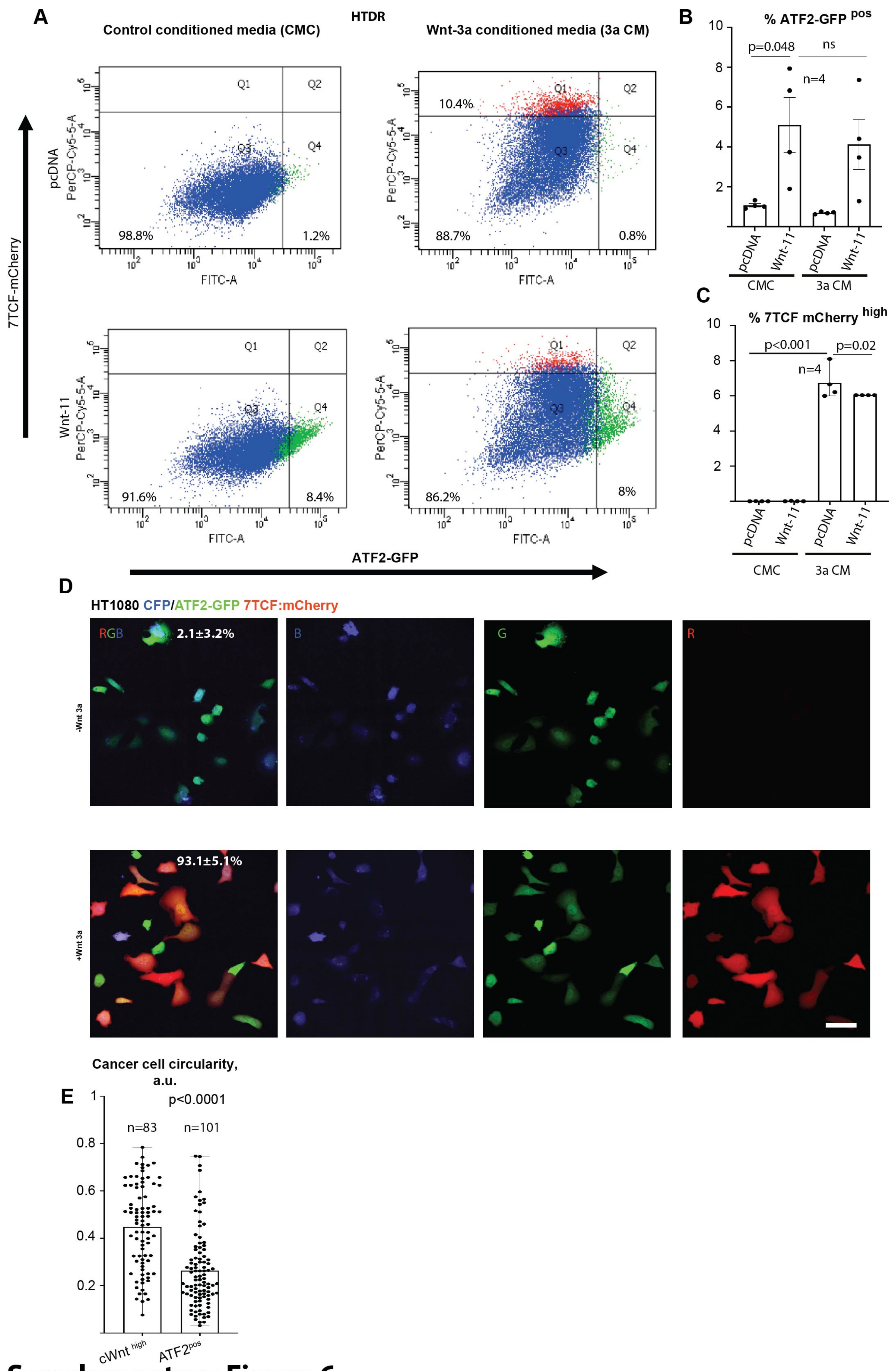

Supplementary Figure 6

Journal of Cell Science • Supplementary information

Fig. S6. ***In vitro*** **characterisation of HTDR double reporter cell line.** (**A**) Representative FACS plots of HDTR cells showing activation of ATF2-GFP (horizontal axis) and 7TCF-mCherry signal (vertical axis) upon expression of either Wnt-11 or empty vector (pcDNA) and treatment with either Wnt-3a-conditioned-media (3a CM) or control conditioned media (CMC); percentages of cells gated are indicated. **B**) ATF2-GFP mean intensity plot for the total population in the cells treated as indicated in (**A**). **C**) 7TCF-mCherry mean intensity plot for the total population in the cells treated as indicated in (**A**). **D)** Representative images showing the response of HTDR cells to stimulation by Wnt-3a (100 ng/ml). Separate panels show red/green/blue (mCherry/Cerulean/GFP) channels, combined or alone. **E)** Cell shape (circularity) differences between cWnthigh and ATF2pos cells. Statistical comparison (unpaired t-test **(B, C)** or Mann-Whitney test **(E)**). n animals = 8 **(E)**. Scale bars = 50 µm.

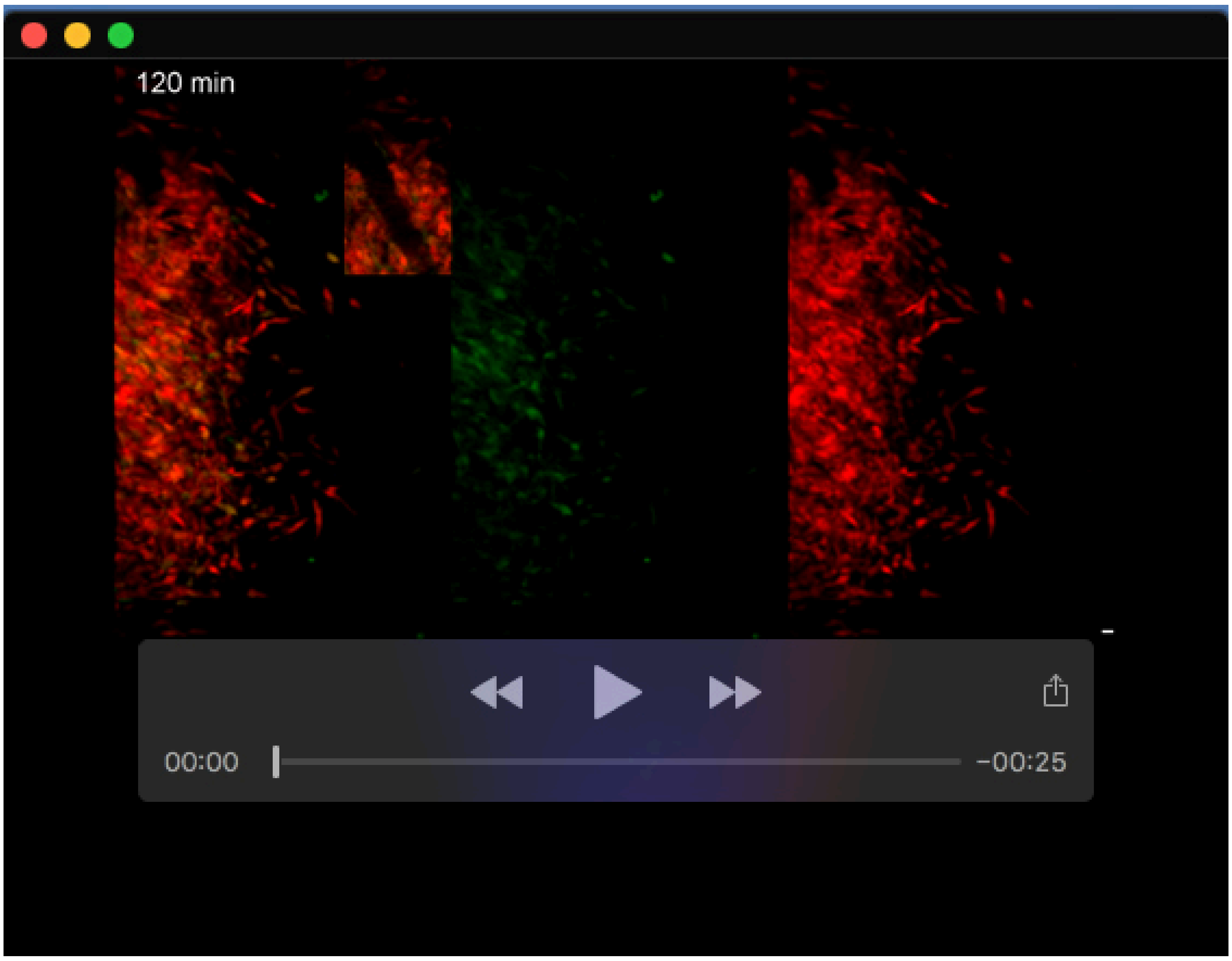

Movie 1. Visualisation of canonical Wnt pathway activity during HT1080 primary tumour cell invasion. Time-lapse video showing cWnt pathway activity in HT1080 TOP Venus reporter-expressing cancer cells during primary tumour cell invasion. Inset shows HT1080 cell intravasation into the blood vessel in the upper right part of the tumour mass. Green arrow points to an HT1080 cell undergoing cWnt^{low} to cWnt^{high} transition. Red arrow points to an HT1080 cell undergoing cWnt^{high} to cWnt^{low} transition. Maximum intensity (3D) projection, 20 min/frame, 29 h total duration; Red = tdTomato, Green = Venus; 10x objective; scale bars = 20 µm.

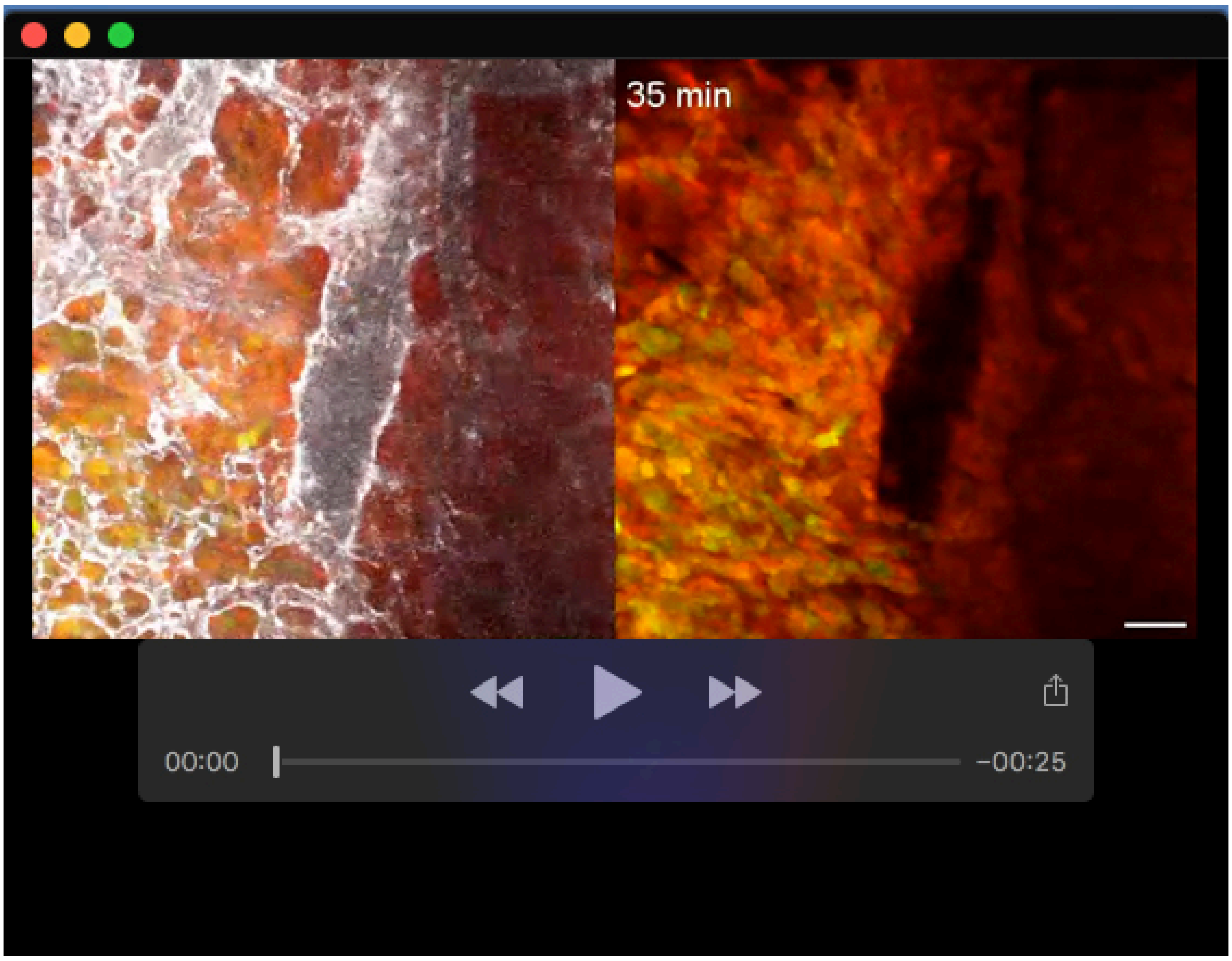

Movie 2. Visualisation of canonical Wnt pathway activity during HT1080 cell intravasation. Representative time-lapse video showing cWnt pathway activity in HT1080 TOP Venus reporter cells during intravasation. White arrows point to an intravasating HT1080 cWnt^{low} cell that is entering the blood stream and rolling along the vascular wall. Maximum intensity (3D) projection, 5 min/frame, 70 min total duration; Red = tdTomato, Green = Venus, White = Lectin 647; 25x objective; scale bars = 50 μm.

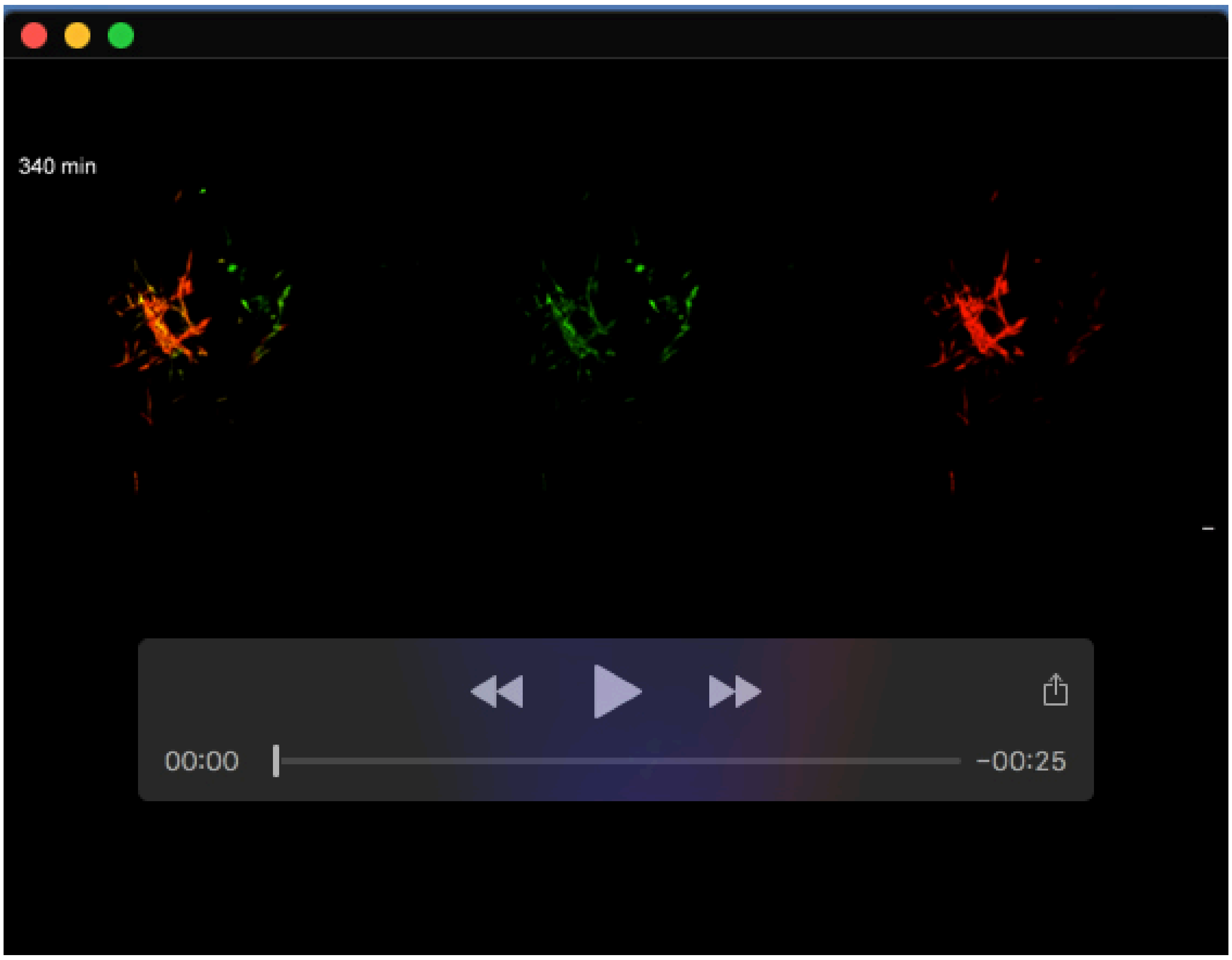

Movie 3. Visualisation of canonical Wnt pathway activity during metastatic lesion invasion. Time-lapse video showing cWnt pathway activity in HT1080 TOP Venus cells during metastatic lesion invasion. Green arrow points to an HT1080 cell undergoing $cWnt^{low}$ to $cWnt^{high}$ transition. Red arrow points to an HT1080 cell undergoing $cWnt^{high}$ to $cWnt^{low}$ transition. Maximum intensity (3D) projection, 20 min/frame, 33 h 20 min total duration; Red = tdTomato, Green = Venus; 10x objective; scale bars = 20 μm.

Journal of Cell Science • Supplementary information

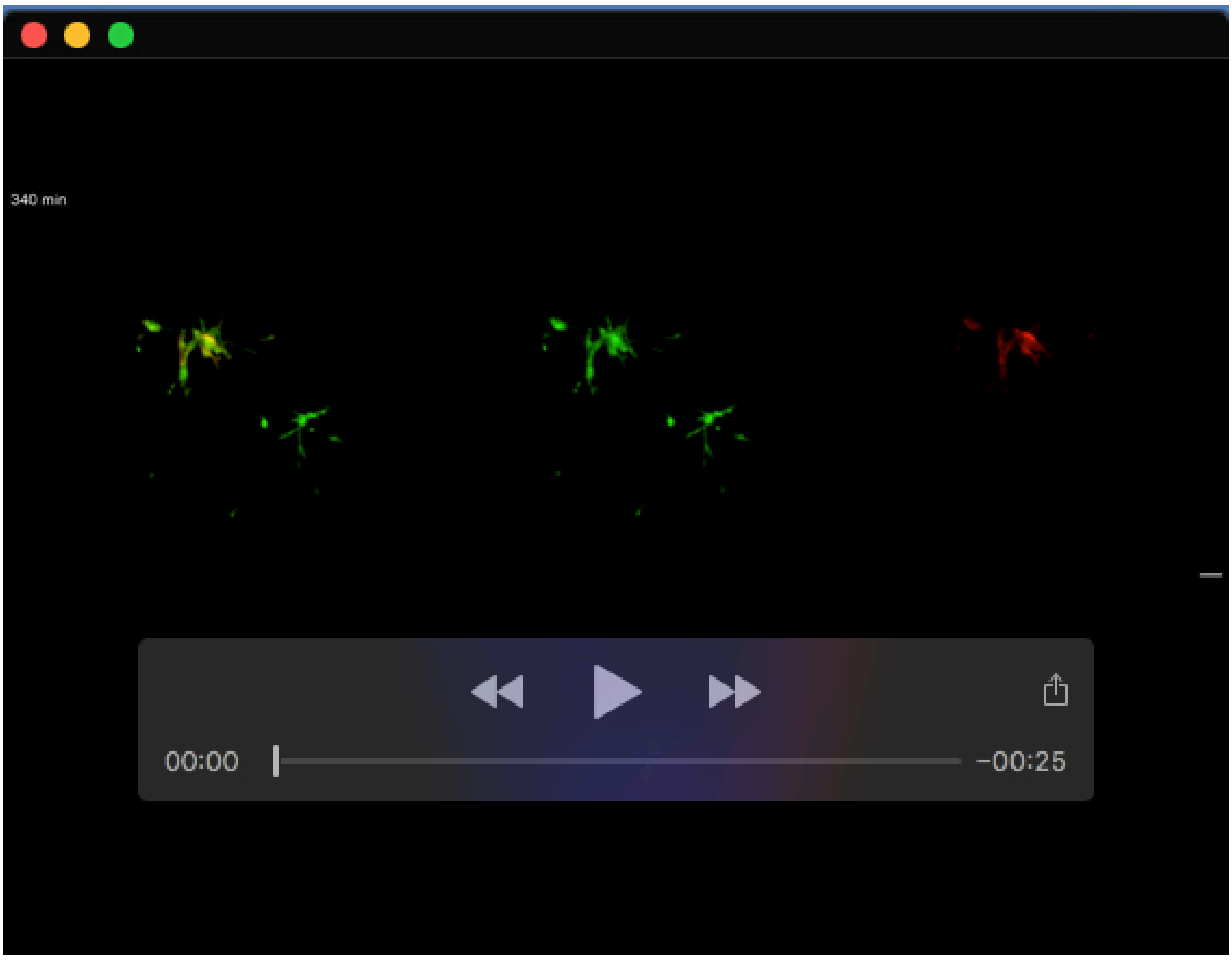

Movie 4. Visualisation of Wnt/β-catenin and ATF2-dependent signalling activities during HTDR cell invasion within metastatic lesions. Time-lapse video showing Wnt/β-catenin- (red) and ATF2-dependent (green) reporter activities during HTDR cell metastatic lesion invasion. Maximum intensity (3D) projection, 20 min/frame, 33 h total duration; Red = mCherry, Green = GFP; 10x objective; scale bars = 50 μm.

Journal of Cell Science • Supplementary information

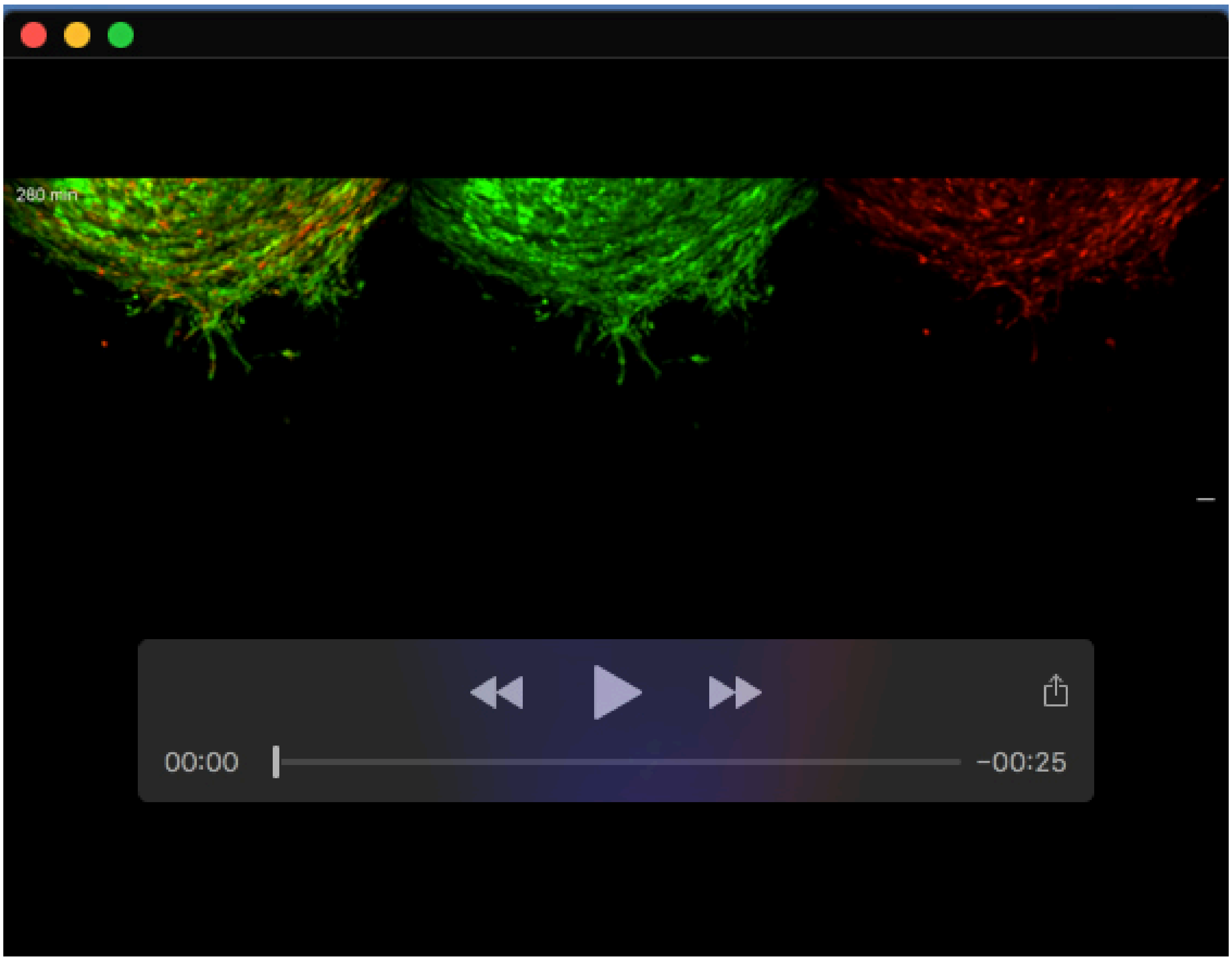

Movie 5. Visualisation of Wnt/β-catenin and ATF2-dependent signalling activities during HTDR cell invasion within the primary tumour. Time-lapse video Wnt/β-catenin- (red) and ATF2-dependent (green) reporter activities during HTDR cell metastatic lesion invasion. Maximum intensity (3D) projection, 20 min/frame, 32 h total duration; Red = mCherry, Green = GFP; 10x objective; scale bars =50 μm.
